# High-Throughput Cloning of Temperature-Sensitive *Caenorhabditis elegans* Mutants with Adult Syncytial Germline Membrane Architecture Defects

**DOI:** 10.1534/g3.115.021451

**Published:** 2015-08-26

**Authors:** Josh Lowry, John Yochem, Chien-Hui Chuang, Kenji Sugioka, Amy A. Connolly, Bruce Bowerman

**Affiliations:** Institute of Molecular Biology, University of Oregon, Eugene, Oregon 97403

**Keywords:** *C. elegans*, whole genome sequencing, gonad development, membrane architecture, membrane dynamics

## Abstract

The adult *Caenorhabditis elegans* hermaphrodite gonad consists of two mirror-symmetric U-shaped arms, with germline nuclei located peripherally in the distal regions of each arm. The nuclei are housed within membrane cubicles that are open to the center, forming a syncytium with a shared cytoplasmic core called the rachis. As the distal germline nuclei progress through meiotic prophase, they move proximally and eventually cellularize as their compartments grow in size. The development and maintenance of this complex and dynamic germline membrane architecture are relatively unexplored, and we have used a forward genetic screen to identify 20 temperature-sensitive mutations in 19 essential genes that cause defects in the germline membrane architecture. Using a combined genome-wide SNP mapping and whole genome sequencing strategy, we have identified the causal mutations in 10 of these mutants. Four of the genes we have identified are conserved, with orthologs known to be involved in membrane biology, and are required for proper development or maintenance of the adult germline membrane architecture. This work provides a starting point for further investigation of the mechanisms that control the dynamics of syncytial membrane architecture during adult oogenesis.

The adult *Caenorhabditis elegans* hermaphrodite reproductive system consists of two mirror symmetrical U-shaped tubular gonad arms that each terminate in a spermatheca and share a common central uterus (Hirsh *et al.* 1976; Kimble and Hirsh 1979) (Figure 1). The gonad arms comprise somatic sheath cells that house a proliferative germline with a complex membrane architecture. At the distal end of each gonad arm, the germline proliferates mitotically. As germline nuclei move proximally and enter meiosis, they adopt peripheral positions, such that the nuclei reside within individual membrane cubicles that are open to and continuous with a shared core cytoplasm called the rachis. As these syncytial meiotic germline nuclei move more proximally, they eventually cellularize and enlarge to become mature oocytes. Although the cellular anatomy of the adult reproductive system and the cell lineages that gives rise to the somatic and germline structures during larval development have been described in detail (Kimble and Hirsh 1979; Hubbard and Greenstein 2000; Kimble and Crittenden 2007; Wong and Schwarzbauer 2012), the membrane dynamics that mediate the movement of syncytial oocyte nuclei and their eventual cellularization during oogenesis in the adult gonad remain poorly understood.

**Figure 1:**
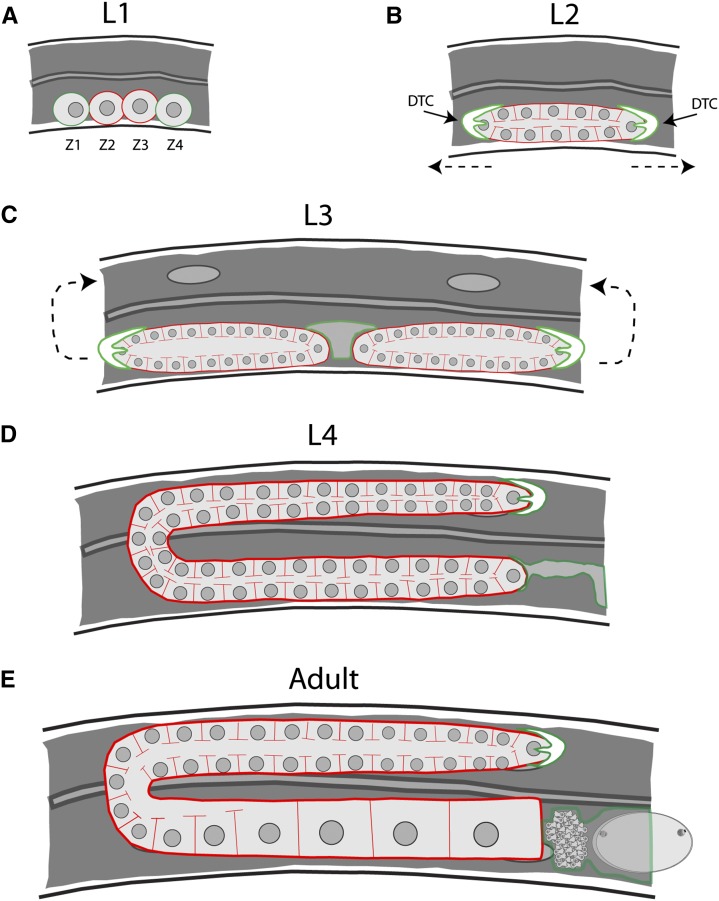
Germline morphogenesis in *C. elegans*. In each panel, germ cells are outlined in red and somatic cells/tissues are in green. (A) At the L1 stage, there are four cells that will give rise to the entirety of the gonad. Two of these cells, Z1 and Z4, will give rise to the somatic tissues of the gonad, whereas Z2 and Z3 will generate the germline lineage. (B) Proliferation of the germline begins after the transition to the L2 stage. This is also the stage in which the germline syncytium forms. The direction of distal tip cell (green outline) migration is denoted by the dashed arrows. (C) As proliferation continues during the L3 stage, the single gonad tube is bisected by the developing somatic gonad tissues, giving rise to two mirror symmetrical arms. The distal tip cell migrates first ventrally, then back toward the center of the animal (arrows). (D) By the L4 stage, the gonad has mostly assumed its final shape, and spermatogenesis begins. Only one of the two arms is shown, with the proximal arm connected to the developing spermatheca and uterus (green outline). (E) Oogenesis begins after the transition to adulthood and will happen continuously throughout the lifespan of the animal.

During embryogenesis, four cells called Z1–Z4 are produced such that they align along the anterior–posterior axis and abut the ventral epidermis of the first stage (L1) larva; these four cells then proliferate to form the adult gonad (Figure 1). The two outermost cells, Z1 and Z4, undergo cell divisions during larval development to produce the somatic structures, which include two distal tip cells (DTCs), gonadal sheath cells, two spermathecae, and the uterus (Kimble and Hirsh 1979). The two innermost cells, Z2 and Z3, proliferate during larval and adult development to produce the germline, including approximately 300 sperm that are made late in larval development, and subsequently all of the oocytes produced by an adult hermaphrodite.

Beginning midway through the L1 larval stage, Z1 and Z4 give rise to 12 descendants that, by the early second (L2) larval stage, form a short tube-like structure in which the germline precursor cells reside (Figure 1). These initial descendants include two distal tip cells, one located at each end of the initial tube-like structure. During the third (L3) larval stage, the somatic gonad precursors undergo additional cell division, and the DTCs migrate in opposite directions along the anterior–posterior axis. The DTCs eventually turn to migrate dorsally, and then turn back toward the center of the worm and migrate back toward each other. Concurrent with DTC migration, gonadal sheath cells migrate and elongate to envelop nearly the entire structure. Morphogenesis is mostly complete by the last (L4) larval stage, when the DTCs reach the center of the animal and stop migrating soon after they meet. As a result, the adult gonad has two U-shaped arms joined centrally by the two spermathecae and the uterus.

Proliferation and development of the germline also take place as the somatic gonad develops. The germline precursors Z2 and Z3 begin to proliferate during the L1 larval stage, and proliferation is subsequently maintained locally by Notch signals from the DTCs. The dividing germline cells undergo incomplete cytokinesis as they proliferate, a process that is regulated by two annilins, ANI-1 and ANI-2 (Maddox *et al.* 2005; Amini *et al.* 2014). This ultimately creates the shared cytoplasmic germline core (the rachis) that is maintained throughout adulthood. As the germline stem cell nuclei nearest to the DTCs continue to proliferate, descendant nuclei move away and enter meiosis as they leave the influence of Notch signaling. During adult oogenesis, the distal germline continues to proliferate. As the syncytial meiotic nuclei reach the turn of the gonad arm, all meiotic nuclei either undergo programmed cell death or adopt the oocyte fate. The surviving nuclei then begin to cellularize and mature, greatly increasing in size due in part to actomyosin-dependent cytoplasmic streaming (Wolke *et al.* 2007). The processes of cytoplasmic streaming and oocyte growth and maturation are regulated by at least two signals. One emanates from sperm at the proximal end of the gonad and the other emanates from the DTCs at the distal end (Govindan *et al.* 2009; Nadarajan *et al.* 2009). Sperm release the major sperm protein (MSP), a signaling ligand that acts through the somatic gonad sheath cells and cyclic AMP to promote oocyte growth and maturation (Govindan *et al.* 2009). The DTCs limit oocyte growth through Notch signaling in a process that appears to occur independently of the DTC Notch signaling that promotes mitotic germline proliferation in the distal gonad (Nadarajan *et al.* 2009). Together, these two signals coordinately regulate oocyte growth. Finally, the most mature oocytes complete cellularization. They close off from the rachis before reaching and ultimately passing through the spermathecae, where they are fertilized before entering the uterus (McCarter *et al.* 1999; Maddox *et al.* 2005). In young adult hermaphrodites, ovulation occurs approximately every 23 min, producing in 6.5 hr zygotes approximately equal in volume to the entire body of the worm (Hirsh *et al.* 1976; McCarter *et al.* 1999; Wolke *et al.* 2007). Thus, the hermaphrodite gonad functions like a conveyor belt, with germline nuclei traveling down the length of the arm as they proceed through meiosis, eventually cellularizing, enlarging, and maturing prior to fertilization upon ovulation.

The complex membrane architecture of the adult germline and its ability to produce large numbers of oocytes throughout the adult life span imply a requirement for extensive and dynamic regulation of membrane production and trafficking. Consistent with such a requirement, the v-SNARE SNB-1 localizes to the plasma membrane in the distal mitotic region of the gonad, and loss of function of early secretory pathway components, such as members of the COPII complex, result in a failure of SNB-1 to localize properly, a failure to form new membrane partitions between germline nuclei following division, and severe disruption of the gonad membrane architecture (Green *et al.* 2011; Hanna *et al.* 2013). In other contexts, the partitions that arise during cellularization in *Drosophila* embryos require the trafficking of recycling endosomes for growth (Lecuit 2004), and requirements for endosome trafficking also have been found for the completion of cytokinesis (Schiel and Prekeris 2013). While an extensive RNAi-based screen has identified many essential *C. elegans* genes required for proper development of the adult gonad (Green *et al.* 2011), very little is known about the regulation of membrane dynamics during adult oogenesis. As the syncytial nature of the germline is widely conserved (Greenbaum *et al.* 2015; Haglund *et al.* 2011), *C. elegans* provides a useful genetic system for investigating how membrane dynamics are controlled during oogenesis.

Membrane trafficking also is required for *C. elegans* eggshell development (Olson *et al.* 2012; Sato *et al.* 2006, 2008). The eggshell has a trilaminar structure and is composed of proteins secreted by the zygote following fertilization, with secreted enzymes also participating in eggshell synthesis. Immediately after fertilization, the outermost vitelline layer forms, creating a block to polyspermy. This is closely followed by activation of CHS-1, an enzyme that synthesizes chitin at the cell surface, forming the second layer of the eggshell. Finally, cortical granules carrying the chondroitin proteoglycans CPG-1 and CPG-2 are secreted while CHS-1 is simultaneously internalized, forming the third layer. Failure of cortical granule exocytosis results in embryos that are defective in eggshell formation and are osmotically sensitive. Some membrane trafficking genes are known to be required for cortical granule formation and exocytosis. For example, loss of function of either *rab-11.1* or the syntaxin *syn-4* results in the aberrant accumulation of caveolin-positive vesicles, normally present at the cortex, around the nuclei of proximal oocytes (Sato *et al.* 2008). Furthermore, loss of *pod-1*, a *C. elegans* coronin homolog, results in osmotic sensitivity due to the misregulation of the timing of cortical granule exocytosis (Rappleye *et al.* 1999; Olson *et al.* 2012).

Given the known requirements for membrane trafficking in both germline development and eggshell formation, we have screened a large collection of temperature-sensitive, embryonic lethal *C. elegans* mutants for defects in both gonad development and eggshell production. These mutants exhibited embryonic osmotic sensitivity when homozygous mutant worms were up-shifted to the restrictive temperature at the L4 larval stage, after the completion of most gonad development. However, when up-shifted to the restrictive temperature at the L1 larval stage, homozygous mutants matured into sterile adults with defects in gonad development. We have identified the causal mutations in a subset of these mutants and present a preliminary analysis of their requirements during gonad development and adult oogenesis.

## Materials and Methods

### *C. elegans* strains

All strains were handled and maintained following standard procedures (Brenner 1974). TS alleles were generated by way of a forward genetic screen using chemical mutagenesis, as previously described (Kemphues *et al.* 1988; Encalada *et al.* 2000; O’Rourke *et al.* 2011a). Following isolation, TS mutant strains were outcrossed to wild-type (N2 Bristol) males to remove the *lin-2(e1309)* mutation present in the parental strain used for screening. TS mutant strains were maintained at the permissive temperature of 15°. Embryonic phenotype characterization was performed by selecting L4 hermaphrodites grown at the permissive temperature and moving them to 26° for a minimum of 6 hr before dissecting embryos into water. The dye permeability assay used to determine permeability barrier defects is described in Carvalho *et al.* (2011). Postembryonic phenotype characterization was performed by moving synchronized populations of L1 larvae to 26° for several (3–5) days and scoring adult germline morphology. Isolated TS alleles and deletion alleles used are listed here by chromosome: *rpl-7(or990*ts*)* I; *rpl-7(or1247*ts*)* I; *abtm-1(or1400*ts*)* I; *abtm-1(tm2721)* I; *or1184*ts I; *or1613*ts I; *or983*ts I; *or819*ts I; *ippk-1(or1572*ts) II; *ippk-1(tm4718)* II; *vps-15(or1235*ts) II; *vps-15(ok3132)* II; *crn-3(or959*ts) II; *crn-3(ok2269)* II; *sqv-8(or888*ts) II; *sqv-8(n2822)* II; *or1112*ts II; *atx-2(or821*ts) III; *atx-2(tm4373)* III; *ndg-4(or1088*ts) III; *ndg-4(sa529)* III; *or1565*ts III; *or1439*ts III; *drp-1(or1393*ts) IV; *drp-1(tm1108)* IV; and *or1233*ts IV. See Supporting Information for a list of mutant strains used for complementation tests to identify causal TS Eos/Ste mutations (Table S2).

### Genetic analysis

Embryonic lethality was scored at both 15° and 26° by singling out 10 L4 hermaphrodites onto individual plates and incubating them at the desired temperature overnight. The following day, the parents were removed and the number of eggs and larvae were counted. On the third day, this count was repeated to determine percent hatching rate. To determine heterozygous hatching rate, mutant adult hermaphrodites were mated to N2 males and the F1 progeny were subjected to the above procedure. Complementation tests were scored in a similar manner, with homozygous TS mutant males being crossed into either a heterozygous, balanced strain carrying a lethal deletion allele or a homozygous viable deletion allele. Brood sizes at the permissive and restrictive temperatures were determined by singling out L4 hermaphrodites and culturing them overnight on individual plates. The following day, each parent was transferred to a new plate, and the number of eggs and larvae were counted. This continued until the parents ceased to produce fertilized eggs.

### Whole-genome sequencing and data analysis

In preparation for sequencing, mutant hermaphrodites were outcrossed to CB4856 males, and ∼200 F2 worms were singled out from the resultant progeny. These were then assayed for homozygous embryonic lethality by picking 10–12 L4 hermaphrodites of the F3 generation from each F2 plate and incubating them at 26° for 3 d. Gravid adults from each homozygous outcross strain were then pooled together and subjected to hypochlorite treatment to destroy any contaminating microbial DNA, using approximately equally sized populations from each strain. The resulting L1s were then used for genomic DNA extraction, which was performed using a Qiagen DNeasy kit. Libraries were prepared for sequencing using Illumina’s Nextera DNA Library Preparation kit, following the included protocol with no modifications. Sequencing was performed on an Illumina HiSequation 2000 at the University of Oregon’s Genomics Core Facility.

All data analysis tools were run using the Galaxy platform (Giardine *et al.* 2005; Goecks *et al.* 2010). The workflow used is the same as that described by Minevich *et al.* (2013), with some modifications. Read mapping was performed using Bowtie2 (Langmead and Salzberg 2012), alignment quality control was performed using a combination of Picard (http://broadinstitute.github.io/picard/) and GATK (DePristo *et al.* 2011), and variant detection and analysis were performed using a combination of GATK, CloudMap, snpEff (Cingolani *et al.* 2012), and Bedtools (Quinlan and Hall 2010). SNP mapping plots were generated using the CloudMap tool.

### CRISPR/Cas-9 generation of GFP::DRP-1 transgenic strain

For *gfp*::*drp-1* donor construct, a 2250-bp fragment (IV: 5537396–5539646) amplified from fosmid WRM069aH08 was cloned into the pJET1.2 vector (ThermoScientic cat. no. K1231). The GFP coding region amplified from pSO26 (O’Rourke *et al.* 2007) was inserted at the N terminus of *drp-1* by Gibson assembly (Gibson *et al.* 2009). A point mutation was then introduced to disrupt the PAM site (CGG to CCG) by inverse PCR. *eft-3p*::Cas-9 plasmid with *drp-1* N terminus targeting sgRNA (GTAGTCGTCGGATCACAGT) was made from pDD162 plasmid (Dickinson *et al.* 2013). The *gfp*::*drp-1* donor construct, Cas-9-sgRNA plasmid, and selection marker pRF4 were injected into wild-type N2 young adults. The animals with correct knock-in were identified by screening for GFP expression (Kim *et al.* 2014).

### Microscopy

Whole worms were prepared for live imaging by anesthetic treatment with 0.5 mg/mL tetramisole-HCl in M9, and were then mounted on a 4% agarose pad and secured with a cover slip. Imaging was performed on a Leica DMI 4000B inverted compound microscope equipped with a Leica 63X/1.40–0.60 HCX Plan Apo oil objective lens. Image capture was accomplished using a Hamamatsu EM-CCD digital camera. Volocity (PerkinElmer Inc.) software was used to control the system. The entire germline was imaged by acquiring multiple stacks along the length of the gonad; each stack consists of 49 *z*-planes with a spacing of 0.5 μm. Visualization and analysis of the stacks were performed using ImageJ (http://imagej.nih.gov/ij/). Composite images were prepared using images from each stack corresponding to the same focal plane, and they were then stitched together using Adobe Illustrator.

### Data availability

All SNP mapping and whole genome sequence data is available at wormbase.org.

## Results

### Identifying conditional mutants that are both eggshell-defective and adult sterile

To identify mutants that might have defects in adult germline membrane dynamics, we scored a collection of ∼800 temperature-sensitive, embryonic lethal (TS-EL) mutants, isolated using a previously described procedure (Kemphues *et al.* 1988; Encalada *et al.* 2000; O’Rourke *et al.* 2011a), for eggshell integrity and fertility defects. To identify eggshell-defective mutants (Eos, for embryonic osmotic sensitivity), we up-shifted homozygous mutant worms that had been raised at the permissive temperature of 15° to the restrictive temperature of 26° at the L4 stage. After these L4 mutant larvae matured to adulthood overnight, early embryos were dissected into sterile water for inspection using DIC microscopy. Embryos with eggshell defects either swell or lyse in a hypotonic environment, and we used this phenotype to qualitatively identify 102 eggshell-defective mutants (J. Lowry, unpublished data). We then further examined this group of mutants for defects in fertility by up-shifting populations of homozygous mutant L1 larvae to 26° and scoring them for fertility after they matured to adulthood at the restrictive temperature. While most of the adult mutants were fertile but produced inviable embryos, for 49 of the mutants we found that ∼50% or more of the up-shifted larvae either arrested during larval development or matured into sterile adults (Ste, for adult sterile; J. Lowry, unpublished data). These 49 Eos/Ste mutants were then selected for further characterization based on the relatively high penetrance of their adult sterility.

We next genetically characterized the mutants to determine if they caused recessive and, hence, reduction of function phenotypes. First, we used wild-type (N2) males to backcross each homozygous Eos/Ste mutant strain. The backcross was done to eliminate the recessive mutation *lin-2(e1309)* from the parental strain used for mutagenesis; *e1309* renders adult hermaphrodites egg-laying defective and thus suitable for the screening procedure used to identify embryonic-lethal mutants (Kemphues *et al.* 1988; Encalada *et al.* 2000; O’Rourke *et al.* 2011a). Then, we again crossed each backcrossed and homozygous Eos/Ste mutant strain with wild-type males and scored embryonic viability in the broods produced by self-fertilization after up-shifting heterozygous F1 (ts/+) L4 stage worms to the restrictive temperature. We observed 5% or less embryonic lethality in 20 mutants that we selected for further analysis, reasoning that most of these mutants likely carry recessive loss-of-function mutations, with embryonic lethality at levels similar to that observed when wild-type worms were up-shifted to 26° as L4 larvae. We also scored embryonic lethality for the F2 self-progeny produced by heterozygous F1 ts/+ worms raised at the permissive temperature of 15°, after shifting the F2 self-progeny to the restrictive temperature as L4s. In all 20 cases, we found that ∼25% of the F2 progeny produced dead embryos (unpublished data), consistent with the mutants carrying recessive, loss-of-function mutations in a single locus. We then quantified embryonic lethality and adult sterility in homozygous backcrossed mutants at both the permissive (15°) and restrictive (26°) temperatures, and quantified the eggshell-defective phenotypes in embryos produced at the restrictive temperature using a dye permeability assay (Table 1). We then used whole genome sequencing in an effort to identify the causal mutations, as described below.

**Table 1:** Embryonic lethality, adult sterility, and eggshell permeability for temperature-sensitive mutants

Allele	Embryonic Lethality (15°C)	Embryonic Lethality (26°C)	Heterozygous Embryonic Lethality (26°C)	Permeability Barrier Defects (26°C)	Adult Sterility (15°C)	Adult Sterility (26°C)
*or819*ts	15.8% (336)	92.7% (55)	0.52% (191)	16.3% (92)	0% (128)	Larval arrest
*atx-2 (or821*ts)	37.4% (388)	99.6% (715)	8.3% (144)	99.4% (160)	2.2% (137)	86% (122)
*sqv-8 (or888*ts)	1.4% (422)	99.7% (311)	2.0% (298)	92.5% (280)	2.8% (141)	100% (127)
*crn-3 (or959*ts)	4.8% (124)	74.6% (405)	2.6% (288)	68.3% (63)	1.6% (129)	100% (107)
*or983*ts	1.3% (224)	98.9% (352)	1.1% (266)	14.3% (133)	0% (112)	Larval arrest
*rpl-7 (or990*ts)	0.5% (551)	93.8% (130)	2.9% (450)	34.2% (73)	1.4% (140)	100% (113)
*ndg-4 (or1088*ts)	16.5% (375)	84.9% (179)	4.5% (220)	4.6% (307)	1.2% (166)	100% (102)
*or1112*ts	0.5% (394)	100% (837)	2.7% (296)	59.0% (122)	0% (117)	Larval arrest
*or1184*ts	11.6% (431)	100% (290)	33.3% (303)	0% (169)	0% (128)	100% (108)
*or1188*ts	15.8% (275)	88.1% (109)	4.9% (346)	1.6% (187)	2.3% (128)	61% (101)
*or1233*ts	22.1% (412)	100% (319)	5.2% (173)	89.7% (261)	14.3% (112)	50% (121)
*vps-15 (or1235*ts)	1.5% (275)	100% (117)	3.6% (446)	3.4% (237)	0.9% (107)	Larval arrest
*or1247*ts	0.5% (412)	97.5% (81)	1.8% (325)	43.4% (122)	1.8% (112)	100% (95)
*or1353*ts	17.3% (249)	83.1% (355)	5.6% (195)	63.6% (110)	0% (133)	55% (98)
*drp-1 (or1393*ts)	56.6% (571)	99.7% (294)	1.1% (363)	55.2% (145)	0.6% (160)	45% (135)
*abtm-1 (or1400*ts)	3.3% (271)	100% (181)	3.9% (442)	55.2% (375)	1.2% (167)	100% (121)
*or1409*ts	2.2% (543)	100% (225)	3.5% (310)	46.8% (252)	0% (169)	100% (113)
*or1439*ts	2.9% (452)	91.6% (143)	1.5% (456)	23.8% (168)	0.9% (116)	83% (105)
*or1565*ts	45.4% (280)	90.2% (102)	10% (289)	78.7% (389)	65.2% (125)	100% (111)
*ippk-1 (or1572*ts)	9.5% (494)	100% (465)	1.1% (366)	3.8% (366)	17.8% (185)	Larval arrest
*or1613*ts	0.03% (324)	97.2% (253)	1.1% (190)	42.8% (180)	0% (117)	100% (119)
*or1621*ts	3.1% (478)	100% (496)	4.1% (168)	97.4% (651)	0% (116)	58% (103)

Embryonic lethality and adult sterility for homozygous mutants were scored at the permissive (15°C) and restrictive (26°C) temperatures. Embryonic lethality for heterozygous mutants was scored at the restrictive temperature. Eggshell permeability barrier defects for homozygous mutants were scored at the restrictive temperature using a dye permeability assay (Carvalho *et al.* 2011).

### Identifying the causal mutations in Eos/Ste mutants using whole genome sequencing

**Figure 2:**
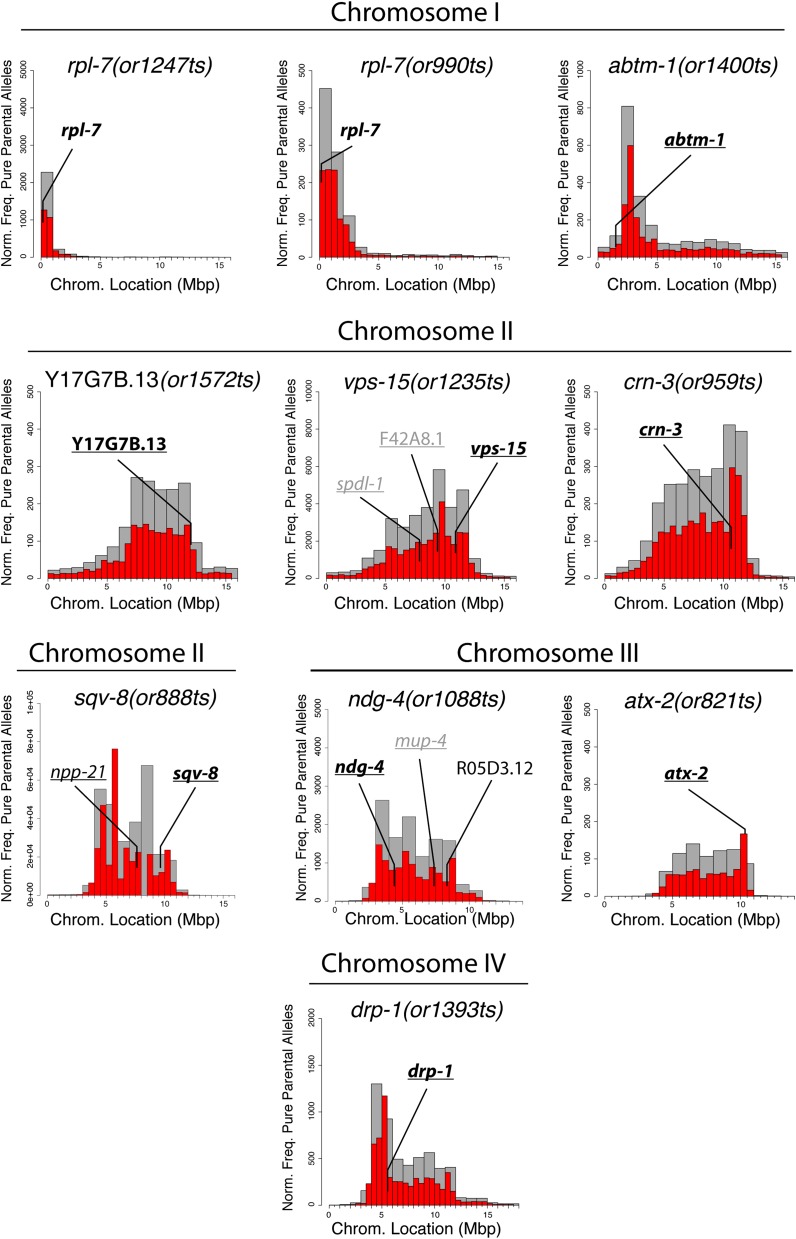
SNP mapping data for temperature-sensitive mutations with identified causal mutations. For each mutant, the frequency of homozygous parental alleles was plotted against chromosomal position in bins of either one megabase (gray bars) or half-megabase (red bars). Gene names on each plot are essential genes in which mis-sense mutations were detected. Underlined gene names are those for which deletion alleles exist for performing complementation tests. For complementation tests in which the mutations failed to complement each other, the gene names are in bold; for tests in which the mutations complemented, the gene names are in gray.

Because mutations in many essential genes required more generally for gene expression or metabolism might indirectly lead to both eggshell defects and larval arrest or sterility, we sought to identify the causal mutations in our set of 20 TS Eos/Ste mutants using a whole genome sequencing strategy before further examining the mutant phenotypes. Chemically mutagenized *C. elegans* strains have been shown previously to have hundreds of genomic mutations relative to the reference wild-type genome sequence, even after multiple rounds of backcrossing (Sarin *et al.* 2010). We therefore used an approach that provides both genome-wide SNP mapping and genome sequence data following a single outcross to a polymorphic Hawaiian strain called CB4856 (Doitsidou *et al.* 2010). In brief, we crossed CB4856 males to homozygous Eos/Ste hermaphrodites at the permissive temperature and then isolated 20–50 homozygous F2 animals scored for embryonic lethality after L4 up-shifts of F3 progeny to the restrictive temperature. We then pooled together for each mutant several individuals from the homozygous (ts/ts) F3 and F4 generations for genomic DNA isolation, and sequenced each pool using paired-end Illumina DNA sequencing (see *Materials and Methods*). This generated a pool of recombinant chromosomes with a shared lack of introduced CB4856 polymorphisms in the region linked to the causal mutation. This trait can be detected computationally using the CloudMap tool (Minevich *et al.* 2012), providing genetic mapping data. More specifically, the regions most tightly linked to the causal mutations often have the highest frequency of pure parental alleles (Figure 2). Previous work that applied this approach to viable mutants found that most causal mutations mapped to within 1 Mb of the highest peak in plots of pure parental allele frequency (Doitsidou *et al.* 2010, Minevich *et al.* 2012). We therefore used this approach to narrow the genomic regions of interest for these 20 TS Eos/Ste mutations.

After using pure parental frequency to estimate the location of Eos/Ste mutations in the genome, we then filtered the sequence data for candidate mutations within the region of interest (see *Materials and Methods*). Our previous work with other TS-EL mutants has shown that most conditional alleles are mis-sense mutations, and less frequently splice-site mutations, in genes that are known to be essential based on genome-wide RNAi screens (O’Rourke *et al.* 2011a). For each Eos/Ste allele, we generated from the whole genome sequence data short lists of one to five candidate genes found within several megabase intervals derived from the SNP mapping data, focusing on mis-sense and splice site mutations in essential genes. We then used complementation tests, when mutant alleles were available, to identify the causal mutations. For eight of the mutants, the Eos/Ste alleles failed to complement known alleles of candidate genes and complemented available deletion alleles for other candidate genes (Table S1 and Table S2). For two mutants, *or990*ts and *or1247*ts, a different mis-sense mutation was found in the same gene in both mutants; this was the only candidate gene shared by both mutants, and the two mutants failed to complement each other (Table S1). Based on these mapping and complementation test data, we have identified the causal mutations in 10 Eos/Ste strains, as described in more detail below. For an additional eight mutants, we have narrowed the candidate genes to one or two loci, but deletion alleles of those genes do not exist, precluding complementation tests that could identify the causal mutation. Finally, in two cases, the TS alleles complemented deletion alleles for all candidate genes (J. Lowry, unpublished data), indicating that in some cases additional analysis will be needed to identify causal mutations (see *Discussion*).

### Mutational inactivation of genes with general roles in gene expression or metabolism can cause the Eos/Ste phenotype

**Figure 3:**
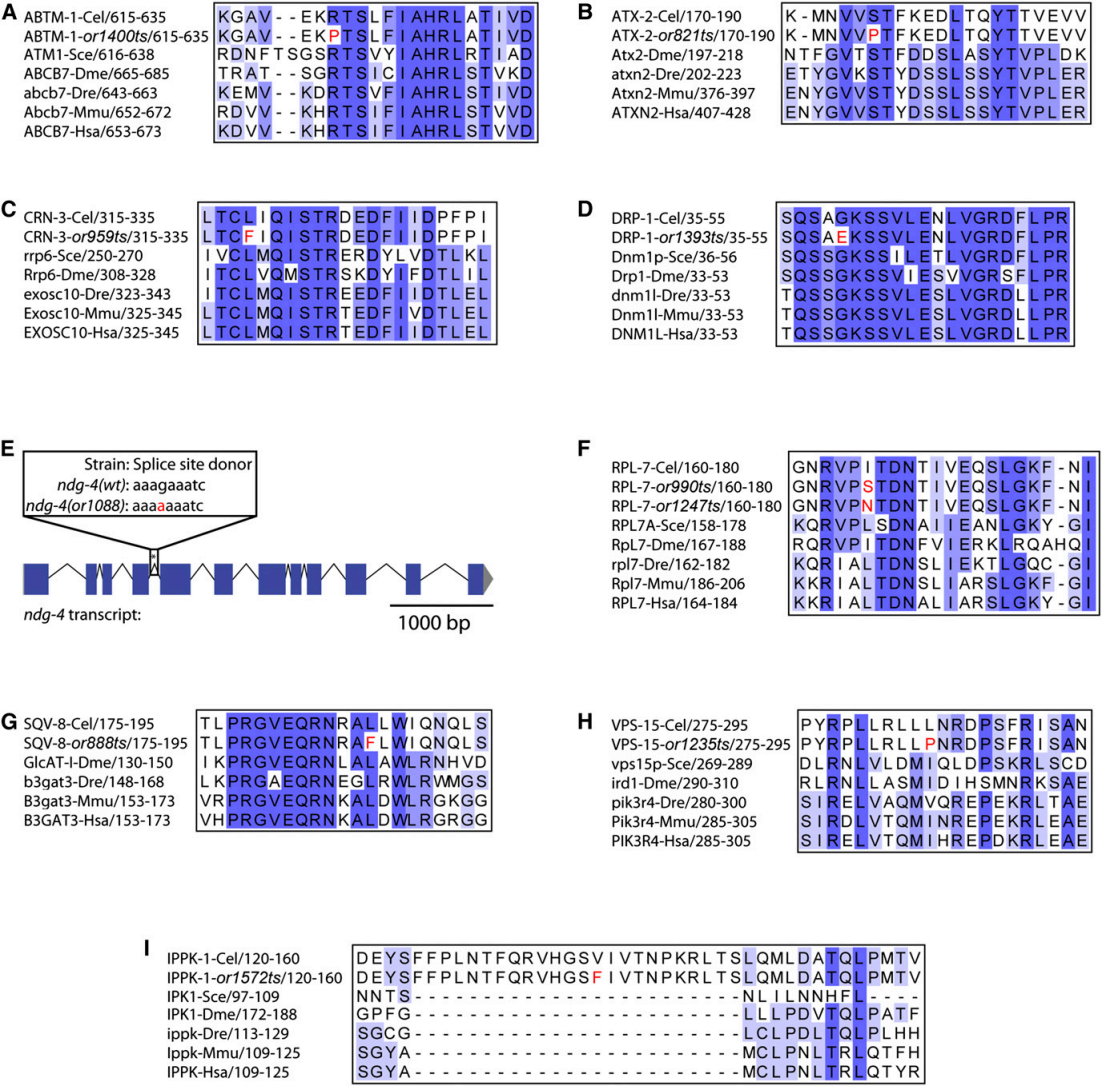
Conservation of amino acids for identified causal mutations. (A–D, F–I) For each conserved gene, both the wild-type and mutant *C. elegans* protein sequences were aligned against homologs from other species (Cel = *C. elegans*; Sce = *S. cerevisiae*; Dme = *D. melanogaster*; Dre = *D. rerio*; Mmu = *M. musculus*; Hsa = *H. sapiens*). The level of sequence conservation has been highlighted using Jalview’s Color by Percentage Identity function (Waterhouse *et al.* 2009). (E) Schematic of the *ndg-4* transcript, with the mutated splice donor site highlighted with an asterisk (*).

For all but one of the 10 Eos/Ste mutants in which we have identified causal mutations, the affected genes are conserved across vertebrate species (Figure 3), with published data providing some insight regarding their possible roles in *C. elegans* (see *Discussion*). While our goal has been to identify genes that are important for membrane dynamics in the adult gonad during oogenesis, we anticipated that mutations in genes required more generally for metabolism and gene expression, without being directly involved in the regulation of membrane dynamics, might also cause both eggshell defects and adult sterility after L1 and L4 up-shifts, respectively. For example, RNAi knockdown screens have found that genes encoding ribosomal proteins are required both for eggshell integrity and for adult fertility (Green *et al.* 2011; Sönnischen *et al.* 2005; Kamath *et al.* 2003) .

Consistent with our expectation that our screening procedure would identify more generally required genes, we have identified two alleles of *rpl-7*, *or990*ts and *or1247*ts, which encode a ribosomal protein subunit required for rRNA processing (Jakovljevic *et al.* 2012). We also have identified one allele of *abtm-1*, *or1400*ts, which encodes an ABC family iron transporter active in the mitochondrial inner membrane (Zutz *et al.* 2009); one allele of *crn-3*, *or959*ts, which encodes an RNA endonuclease subunit of the exosome complex (Parrish and Xue, 2003; Chlebowski *et al.* 2013); and one allele of *atx-2*, *or821*ts, which encodes an RNA-binding protein that regulates translation in the *C. elegans* germline and is known to be required for eggshell formation (Maine *et al.* 2004; Ciosk *et al.* 2004; Sönnischen *et al.* 2005). We suspect that these genes only indirectly influence membrane dynamics during gonad development and adult oogenesis.

### Some Eos/Ste genes are more directly implicated in membrane biology

Five genes we have identified may more directly impact membrane architecture and dynamics. We identified *or888*ts as an allele of *sqv-8*, which encodes an enzyme required for glycosaminoglycan synthesis (Herman *et al.* 1999). In *C. elegans*, loss of chondroitin synthesis, which requires *sqv-8* function, leads to cytokinesis defects in the early embryo, possibly due to a loss of extracellular matrix integrity during furrow ingression (Mizuguchi et al., 2003; Izumikawa *et al.* 2004; Olson *et al.* 2012). Thus, it is possible that *sqv-8* similarly influences membrane architecture or dynamics during gonad development. Two genes we have identified appear most likely to more directly influence membrane dynamics. One, *or1235*ts, is an allele of *vps-15*, which encodes a regulator of the lipid kinase VPS-34 that, based on studies in yeast and vertebrates, is required for both endocytosis and autophagy (Backer 2008). Another, *or1393*ts, is an allele of *drp-1*, which encodes a dynamin-related protein required for mitochondrial membrane fission (Otera *et al.* 2013). Finally, two of the identified genes are involved in lipid metabolism. One, *or1572*ts, is an allele of Y17G7B.13, which is homologous to human IPPK, a kinase required for the production of inositol 6-polyphosphate (Tsui and York 2010). Based on this homology, we have named the *C. elegans* gene *ippk-1*. The second, *or1088*ts, is an allele of *ndg-4*, which encodes a transmembrane protein that is involved in the transport of dietary lipids into the germline. While homologs of *ndg-4* have not been described in vertebrates, an ortholog in *Drosophila* is important for longevity (Brejning *et al.* 2014).

### Germline membrane architecture defects in adult Eos/Ste mutants after temperature up-shifts during larval development

To investigate whether the genes we identified influence gonad membrane morphology, we next examined adult gonad membrane architecture in live mutants expressing a PH domain fused to mCherry to mark the plasma membrane and Histone2B fused to GFP to mark chromosomes after shifting TS Eos/Ste mutants from the permissive to the restrictive temperature during larval development. We focused most of our analysis on four widely conserved genes that appeared likely to most directly influence membrane dynamics: *sqv-8*, *vps-15*, *drp-1*, and *ippk-1* (see Figure S2 for a more limited analysis of *abtm-1*, *atx-2*, *crn-3*, and *rpl-7* mutants). Homozygous mutant worms were grown at the permissive temperature (15°) and then shifted to the restrictive temperature (26°) at either the L1 or the L4 stage until they matured into young adults that were then mounted for imaging using spinning disc confocal microscopy (see *Materials and Methods*).

**Figure 4:**
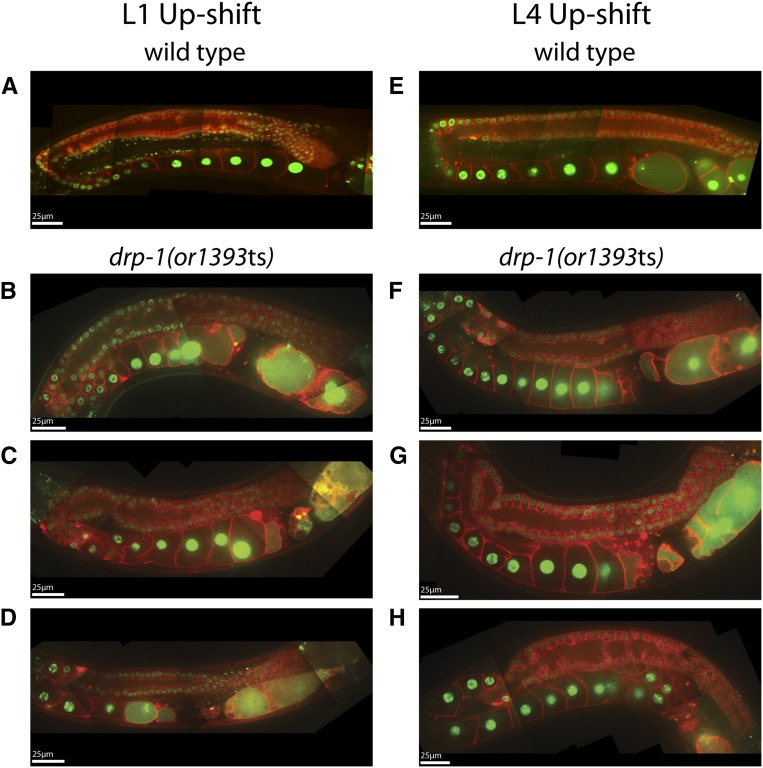
Adult germline defects in *drp-1(or1393*ts) mutants following temperature up-shifts to the restrictive temperature (26°C) at the L1 and L4 larval stages. Spinning disk fluorescent confocal microscopy was used to make composite images of the adult hermaphrodite gonads in both wild-type (A and E) and *drp-1(or1393*ts) (B–D and F–H) worms. In this and subsequent figures, plasma membranes were marked with an mCherry::PH domain fusion, chromosomes were marked with a histone2B::GFP fusion (see *Materials and Methods*), and scale bars are shown in white.

**Figure 5:**
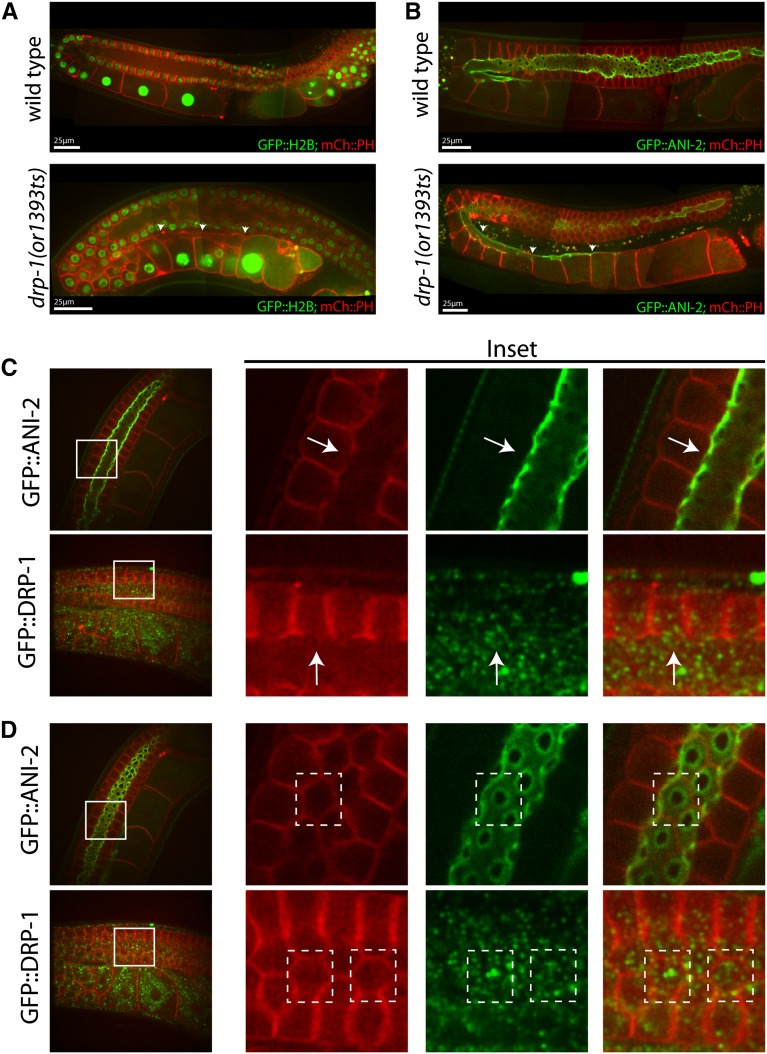
Rachis extension in *drp-1(or1393*ts*)* mutants and GFP::DRP-1 localization. (A) The rachis appears to extend to the most mature oocyte (arrowheads) in an adult *drp-1(or1393*ts*)* mutant gonad, as detected using mCherry::PH to mark the membranes. (B) ANI-2::GFP localizes to the surface of the rachis (Maddox *et al.* 2005), which terminates just after the turn in wild-type hermaphrodites. The rachis again extends proximally in an adult *drp-1(or1393*ts*)* mutant gonad (arrowheads). (C and D) GFP::DRP-1 (see *Materials and Methods*) does not localize to openings to the rachis in the distal gonad or to the rachis more proximally, arguing against a direct role in oocyte cellularization. (C) An orthogonal view of the meiotic germ cell openings to the rachis of wild-type strains carrying either ANI-2::GFP or GFP::DRP-1. GFP::DRP-1 does not localize to the germ cell opening (arrows). (D) A view in the plane of the meiotic germ cell openings from the rachis surface. Solid white lines in (C) and (D) mark insets, and dashed white lines indicate individual germ cells with openings to rachis.

The dynamin-related protein mutant, *drp-1(or1393*ts*)*, was only weakly penetrant for adult sterility after L1 up-shifts (Table 1), and the adult germline in these mutants was relatively normal after both L1 and L4 up-shifts (Figure 4). We observed the most severe defects in the proximal gonad, with multiple small, membrane-bound compartments often visible near the most mature oocytes (Figure 4, B–D, F–H). These membrane structures may result from defective ovulation, as we observed frequent failures in ovulation in live animals using DIC optics (J. Lowry, unpublished data). In some mutant gonads, the rachis appeared to extend abnormally far into the proximal arm, with the most mature oocyte still connected (Figures 4, F and G, Figure 5, A and B; Figure S3A). We considered the possibility that DRP-1 might be involved in narrowing the rachis during oocyte cellularization, or perhaps in maintenance of the openings of the membrane cubicles to the rachis in the distal gonad. However, because mitochondria have been shown to be abnormally long in *drp-1(tm1108)* mutants (Labrousse *et al.* 1999; Breckenridge *et al.* 2009), and because we have found that a CRISPR/Cas-9 generated GFP fusion to the wild-type *drp-1* locus was expressed only in mitochondria and was not detectable elsewhere in the germline (Figure 5, C and D), we suspect the gonad morphology and eggshell defects may be indirect consequences of abnormal mitochondrial function or morphology.

**Figure 6:**
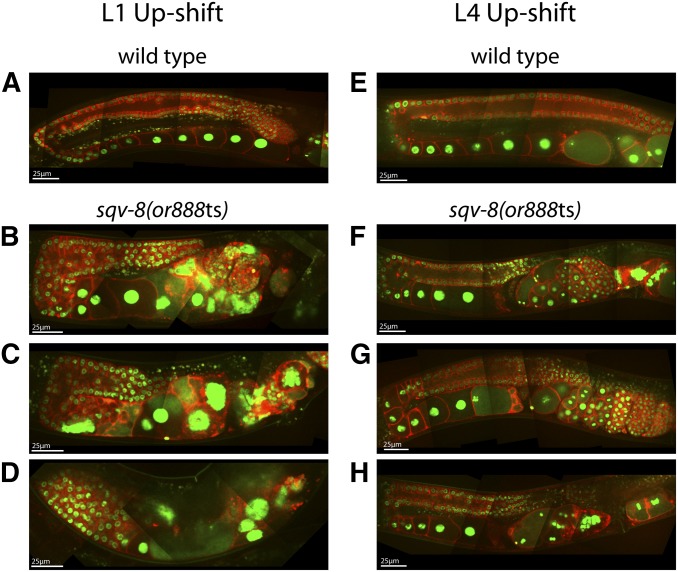
Adult germline defects in *sqv-8(or888*ts) mutants following temperature up-shifts to the restrictive temperature (26°C) at the L1 and L4 larval stages. Composite images were prepared as previously described (Figure 3) for the adult hermaphrodite gonads in wild-type (A and E) and *sqv-8(or888*ts) (B–D and F–H) worms.

We observed severe adult gonad morphology defects following L1 up-shifts of *sqv-8(or888*ts*)* worms. The distal and proximal regions of each arm appeared to be wider than in wild-type, and the arms were shorter in length from the distal end to the spermathecum (Figure 6, B and C). In some cases, the arms consisted of a single wide region with no turn (Figure 6D), and in some animals one gonad arm was small or absent (Figure S3B). The rachis structure in the distal region was severely disrupted, with tightly packed and disorganized germ cell precursors throughout (Figure 6, B–D). The uteri were filled with disorganized membrane and DNA (Figure 6, B–D), perhaps due to the accumulation of burst eggshell-defective embryos within the worms, as *sqv-8* is required for proper vulval development and egg-laying (Herman *et al.* 1999; Herman and Horvitz 1999). The gonad morphology was much more normal after L4 up-shifts, but the adults were still egg-laying defective (Figure 6, F–H).

**Figure 7:**
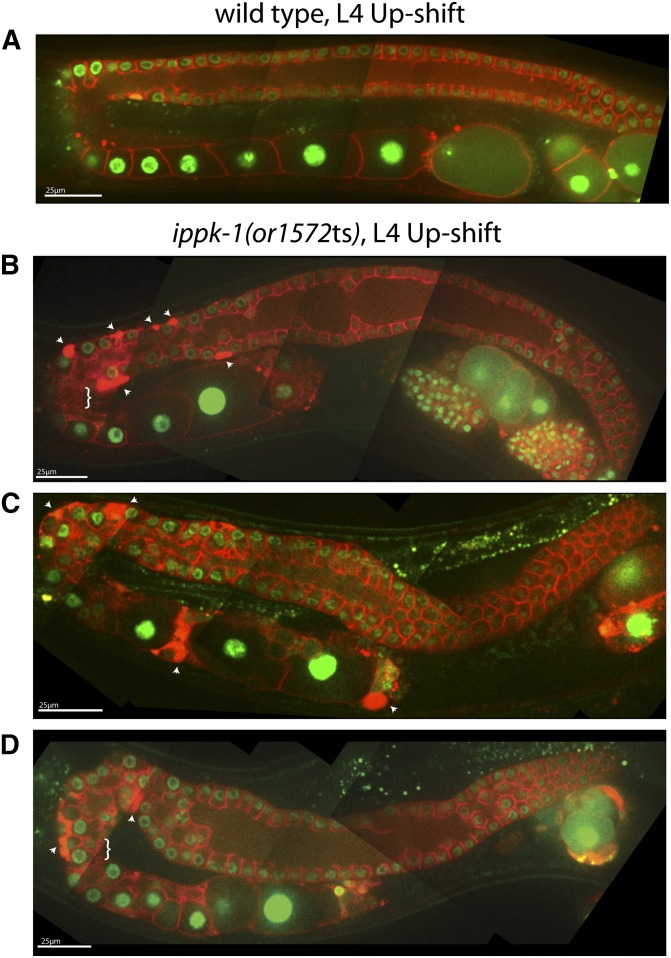
Adult germline defects in *ippk-1(or1572*ts) mutants following temperature up-shifts to the restrictive temperature (26°C) at the L4 larval stage. Composite images were prepared as previously described (Figure 3) for the adult hermaphrodite gonad in wild-type (A) and *ippk-1(or1572*ts) (B–D) worms. Arrowheads point to membrane accumulations in the germline; braces highlight regions of rachis vesiculation.

Because *ippk-1*(*or1572*ts) mutants arrest during larval development after L1 up-shifts, we examined adult gonad morphology only after L4 up-shifts and observed severe abnormalities. The width of the distal region varied along its length, as did the rachis (Figure 7, B–D). We also observed numerous small membrane vesicles near the turn and areas with densely concentrated mCherry::PH signal (arrowheads in Figure 7, B–D). Finally, the distal regions of the gonad arms were in some cases displaced ventrally, rather than lying against the overlying dorsal surface (Figure 7, C and D). This displacement could result from abnormal migration of the distal tip cell during larval development (see *Introduction*), or could be due to a later displacement.

**Figure 8:**
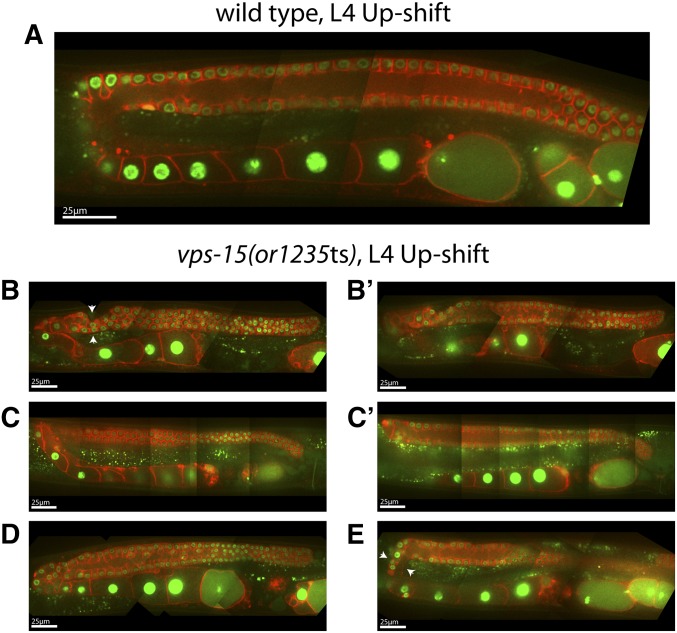
Adult germline defects in *vps-15(or1235*ts) mutants following temperature up-shifts to the restrictive temperature of 26°C at the L4 larval stage. Composite images were prepared as previously described (Figure 3) for the adult hermaphrodite gonad in wild-type (A) and *vps-15(or1235*ts) (B–E) worms. (B and B′, C and C′) Images from the same animal at two different focal planes. Arrows point to regions of constriction at or near the turn of the gonad.

We also observed larval arrest after L1 up-shifts of *vps-15(or1235*ts*)* mutants and thus examined adult gonads for morphology only after L4 up-shifts. The most prominent defect we observed was in the proximal part of the gonad arms, with fewer and often highly elongated oocytes present (Figure 8, B, C, E). The proximal portion of the gonad arms also was abnormally narrow near the turn, with oocytes becoming much wider near the spermathecum (Figure 8, B, D, E). In the distal arm, the rachis appeared abnormally narrow, with a membrane/rachis interface that was less uniform than in wild-type (Figure 8, B′, C′, D, E). The turns of the gonad arms were also abnormally narrow, and we observed abnormal constrictions near the turns (arrowheads in Figure 8, B and E).

### Adult up-shifts reveal genetic requirements for the maintenance of adult germline membrane architecture

**Figure 9:**
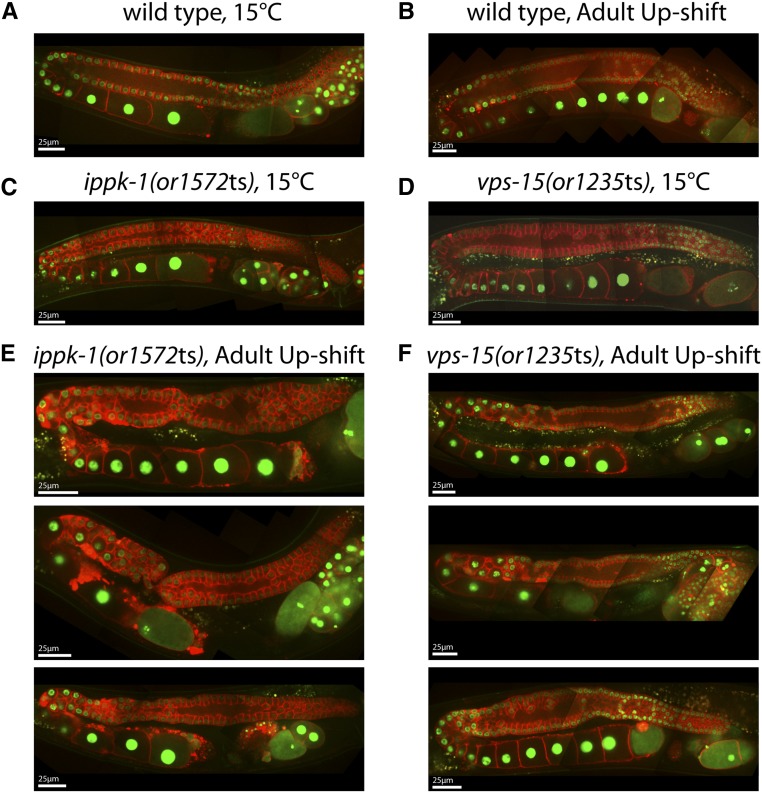
Germline defects in *ippk-1(or1572*ts*)* and *vps-15(or1235*ts*)* mutants after young adult up-shifts to the restrictive temperature (26°C). Composite images of the germline were prepared as previously described for wild-type (A, B), *ippk-1(or1572*ts*)* (C, E), and *vps-15(or1235*ts*)* (D, F) worms. These animals were either grown entirely at 15°C or shifted to 26°C as young adults for 18 hr.

To determine if the genes we have identified are important for maintenance of the gonad membrane architecture during oogenesis in adults, we next examined mutants up-shifted as young adults from the permissive to the restrictive temperature for 18 hr, again using a PH domain fused to mCherry to mark the plasma membrane and GFP fused to Histone2B to mark chromosomes (Figure 9). As a control, we first examined wild-type adults 18 hr after up-shift and found no significant perturbations in gonad membrane morphology (Figure 9, A and B). Similarly, the membrane morphology in adult mutants grown for 18 hr at 15° largely resembled wild-type (Figures 9, C and D; Figure S4C and D). We did not detect significant defects in *drp-1(or1393*ts*)* mutants after adults were fed for 18 hr at the restrictive temperature (Figure S4E), which was not surprising given the minor defects we observed following both L1 and L4 up-shifts. We also failed to detect significant defects in *sqv-8(or888*ts*)* mutants (Figure S4F), even though we had detected severe defects in gonad morphogenesis after L1 and L4 up-shifts (Figure 6). Thus, although *sqv-8* function is important for gonad development, we did not find any *sqv-8* requirements for maintenance of adult gonad membrane morphology.

We did detect membrane architecture defects in both *ippk-1(or1572*ts*)* and *vps-15(or1235*ts*)* mutants after adult up-shifts (Figure 9, E and F), and in both cases the defects resembled those observed after L4 up-shifts. In *ippk-1(or1572*ts*)* mutants, the distal arm varied in width along its length, the mCherry::GFP signal accumulated to high levels in foci near the turns of the gonad arms, and in some animals severe constrictions were formed near the turn (Figure 9E). In *vps-15(or1235*ts*)* mutants, proximal oocyte precursors were disorganized near the turns, and in some animals the oocytes near the turns were narrow and elongated compared to wild-type (Figure 9F). We conclude that *vps-15* and *ippk-1* are required to maintain gonad membrane architecture during adulthood and therefore may be important for regulating adult gonad membrane dynamics during oogenesis.

## Discussion

Although *C. elegans* gonad development has been studied comprehensively, the extensive membrane dynamics that must accompany adult oogenesis have been largely ignored. As the syncytial nature of the germline is widely conserved (Greenbaum *et al.* 2011; Haglund *et al.* 2011), studies of this phenomenon in *C. elegans* are likely to be of general significance. We have therefore attempted to identify genes that mediate membrane dynamics during adult oogenesis by identifying temperature-sensitive, embryonic-lethal (TS-EL) *C. elegans* mutants that are both eggshell-defective (osmotically sensitive) after late larval up-shifts and adult sterile after early larval up-shifts (Eos/Ste mutants), reasoning that both processes involve membrane trafficking. Because general metabolic and gene expression functions might also affect both of these processes, we applied a high-throughput whole genome sequencing strategy to identify the causal mutations in a collection of TS Eos/Ste mutants that all share these two defects. Although we identified alleles of several genes that likely indirectly affect membrane trafficking due to more general roles in gene expression and metabolism, we also identified genes that appear likely to more directly influence membrane dynamics, including two that are required to maintain proper gonad membrane architecture during oogenesis in adult worms. As discussed below, further analysis of these genes may improve our understanding of membrane dynamics during oogenesis. In addition, our identification of TS alleles of other genes that indirectly influence oogenesis provides useful genetic tools for the study of widely conserved gene functions.

### High-throughput identification of temperature-sensitive loss of function alleles of essential *C. elegans* genes

Temperature-sensitive alleles of essential genes are powerful genetic tools, as many essential genes have multiple requirements throughout the life of an organism: with TS alleles, one can bypass early requirements at the permissive temperature and study later requirements after up-shifts to the restrictive temperature (O’Rourke *et al.* 2011a). Moreover, fast-acting TS alleles (that rapidly inactivate and reactivate upon temperature up-shifts and down-shifts) also can be used to define temperature-sensitive periods when their functions are required (Severson *et al.* 2000; Schmidt *et al.* 2005; Davies *et al.* 2014), and TS mutants grown at intermediate temperatures provide sensitized genetic backgrounds for modifier screens that can identify other genes involved in the same processes (Kemp *et al.* 2007; Dorfman *et al.* 2009). The *C. elegans* genome is estimated to contain ∼20,170 protein-coding genes (Shaye and Greenwald 2011), and at least 2500 have essential functions based on genome-wide screens that have used RNA interference to knockdown their functions (Sonnichsen *et al.* 2005; Kamath *et al.* 2003). However, TS alleles have been identified for only a small fraction of these essential genes (we estimate no more than ∼200); therefore, we have explored high-throughput methods for identifying the causal mutations in a large collection of TS-EL mutants (O’Rourke *et al.* 2011b; data reported here).

To identify the causal mutations in the recessive Eos/Ste mutants we isolated, we used a previously developed approach that combines genome-wide single nucleotide polymorphism (SNP) mapping with whole genome sequencing (Jaramillo-Lambert *et al.* 2015; Doitsidou *et al.* 2010; Minevich *et al.* 2013). Because the genome of a single mutant strain isolated after chemical mutagenesis can contain hundreds of mis-sense mutations (Sarin *et al.* 2010), SNP mapping is needed to narrow the interval in which candidate mutations can be found and then further analyzed to identify the causal mutations. Initially developed for homozygous viable mutants, we have modified this procedure for use with TS-EL mutants (see *Materials and Methods*). Using this approach, we were able to identify one to a few candidate mutations (mis-sense mutations, splice site mutations, and mutations in 3′ and 5′ UTRs) within megabase intervals for each mutant (see *Materials and Methods*). To determine if candidate mutations were responsible for embryonic lethality, we used complementation tests with previously isolated mutant alleles, often by taking advantage of deletion alleles that have been isolated by genome-wide knockout consortiums. Of the 20 Eos/Ste mutants we processed for whole genome sequencing, we were able to identify the causal mutations in 10 mutants, based on complementation test results. For eight of the remaining Eos/Ste mutants, deletion alleles were not available for all candidate genes; nevertheless, we were able to narrow down the candidates to one or two loci. For these mutants, we anticipate that CRISPR/Cas-9 technology will make it possible to isolate deletion alleles of the candidate genes and that the causal mutations can then be determined with additional complementation tests. However, for two of the Eos/Ste mutants, deletion alleles were available for all candidate genes, but all of them complemented the TS mutations in genetic crosses. We suspect that these strains harbor mutations that were not captured in our filtering process. For example, they may have mutations in currently uncharacterized genes for which loss of function data are not available, or they may have small deletions that would have been missed with our filtering strategy. Nevertheless, the approach we have used should be able to identify the causal mutations in most TS-EL mutants processed. We plan to systematically apply these methods for high-throughput analysis of large numbers of TS-EL *C. elegans* mutants in an effort to provide a resource of temperature-sensitive mutations for the analysis of essential *C. elegans* gene requirements.

Some progress has been made in computationally predicting amino acid changes that make a protein temperature-sensitive in function (Chakshusmathi *et al.* 2004; Poultney *et al.* 2011; Lockwood *et al.* 2011), and the identification of causal TS-EL mutations from chemical mutagenesis screens may help further guide such efforts. As expected, most (9 of 10) of the mutations we identified are mis-sense mutations; the one exception is *ndg-4(or1088*ts*)*, which alters a splice donor nucleotide (Figure 3 and Table 2). In a previous survey of 24 TS-EL mutations affecting 13 *C. elegans* genes, we similarly found that 20 were mis-sense mutations, two were intronic splice site mutations, one was an in-frame deletion, and one caused a premature stop codon (O’Rourke *et al.* 2011a). The mis-sense mutations we report here most often affected hydrophobic amino acids, changing them to charged or polar amino acids, and five of the nine involve highly conserved residues. Three others affect hydrophobic amino acids that are not fully conserved but are replaced by other hydrophobic residues in different species. Only one, *ippk-1(or1572*ts*)*, affects an amino acid that is not conserved to any extent. The TS-EL mutations that affect conserved amino acids may be useful for engineering conditional mutations in other species (*e.g.*, Schmidt *et al.* 2005).

**Table 2:** Causal mutations for 10 Osm/Ste mutants identified by whole genome sequencing and complementation tests

Allele	Transcript Name	Codon Mutated	Amino Acid Change	Nucleotide Change	Transcript Nucleotide (Spliced)	Average Depth of Coverage
*atx-2 (or821*ts*)*	D2045.1a	175	S > P	T > C	523	75.3X
*sqv-8 (or888*ts*)*	ZK1307.5	187	L > F	C > T	559	24.2X
*crn-3 (or959*ts*)*	C14A4.4	318	L > F	C > T	954	25.6X
*rpl-7 (or990*ts*)*	F53G12.10	165	I > S	T > G	494	30.4X
*ndg-4 (or1088*ts*)*	F56F3.2	—*^a^*	—	C > T	—	25.6X
*vps-15 (or1235*ts*)*	ZK930.1	284	L > P	T > C	851	17.1X
*rpl-7 (or1247*ts*)*	F53G12.10	165	I > N	T > A	494	32.7X
*drp-1 (or1393*ts*)*	T12E12.4	39	G > E	G > A	116	28.3X
*abtm-1 (or1400*ts*)*	Y74C10AR.3	621	R > P	C > G	1862	35.0X
*ippk-1* (*or1572*ts*)*	Y17G7B.13	137	V > F	G > T	409	32.3X

Nature and location of each mutation and the average depth of sequencing coverage are shown for each mutant.

a The mutation in *ndg-4(or1088)* affects a splice site donor at the end of exon IV.

### Identification of genes required for oogenesis

We pursued phenotypic analysis of Eos/Ste TS-EL mutants in an effort to identify genes that are required for the membrane dynamics that mediate oogenesis as oocyte precursors travel from the distal to the proximal end of the gonad and eventually undergo cellularization and enlarge to produce mature oocytes (see *Introduction*). Of the nine genes in which we identified causal mutations, all of them are required for proper gonad development after larval up-shifts, and five of them (*drp-1*, *ippk-1*, *ndg-4*, *sqv-8*, and *vps-15*) have been shown in other studies to be relevant to membrane biology (see *Results*). We did not detect any significant requirements for *drp-1* or *sqv-8* in adults, and we did not investigate *ndg-4* due to a lack of known vertebrate orthologs. However, *vps-15* and *ippk-1* did appear to be required for maintenance of the syncytial gonad membrane architecture in adult worms and thus may be required for membrane dynamics during oogenesis.

The *vps-15* gene was identified in budding yeast due to its requirement for vacuolar (lysosomal) protein sorting and acts in multiple membrane trafficking processes (Backer 2008). It encodes a regulator of *vps-34*, a class 3 phosphatidylinositol-3 kinase (PI3K), and also is required for autophagy (Backer 2008). More recently, it has been shown that a core PI3K complex consisting of Vps15-Vps34-Atg6/Beclin-1 is required for endocytosis. During endosome maturation, membrane lipid composition is altered through the action of lipid kinases. Rab5 localizes to early endosomes and interacts directly with Vps15, which in turn recruits the rest of the PI3K complex. The PI3K complex can then generate phosphatidylinositol-3 phosphate (PI(3)P) in the outer leaflet of the endosome, aiding in the recruitment of PI(3)P-binding proteins that continue the early endosome maturation process (Huotari and Helenius 2011). Interactions between the PI3K complex and other effector proteins such as Atg14L, UVRAG, and Rubicon serve to regulate the formation of autophagosomes in a similar manner (Barth and Köhler 2014).

The *ippk-1* gene has not been studied in *C. elegans* but is homologous to the human inositol 1,3,4,5,6-pentakisphosphate 2-kinase (IPPK). Conserved from yeast to humans, IPPK is an enzyme responsible for the production of inositol-6-phosphate (IP_6_) (Tsui and York 2010). IP_6_ is generated from the sequential addition of phosphate groups to IP_3_, which itself is formed from the cleavage of the lipid phosphatidylinositol 4,5-bisphosphate, a membrane lipid. IP_6_ is found at high intercellular concentrations, in some systems reaching as high as 50 μM (Shears *et al.* 2012). In yeast, IP_6_ is required for mRNA export from the nucleus, whereas in higher eukaryotes it functions in the nonhomologous end joining DNA repair pathway (Monserrate and York 2010). In both cases, IP_6_ acts as a cofactor for enzymes required for these processes. IP_6_ also acts as a building block for the more highly phosphorylated inositol pyrophosphates, which themselves have been implicated in a number of cellular roles, including the control of vesicular trafficking (Wilson *et al.* 2013), and thus IPPK-1 might be important for membrane dynamics in the *C. elegans* germline. The enzyme itself is essential in vertebrates, as homozygous Ippk knockout mice are embryonic lethal (Verbsky *et al.* 2005). Furthermore, the zebrafish homolog, ippk, has been shown to play a role in generating left–right asymmetry in zebrafish (Tsui and York 2010). Given its diverse roles in other organisms, the *or1572*ts allele may be a useful tool for analyzing its requirements in *C. elegans*.

A large-scale RNAi-based screen that classified sterile phenotypes after individually knocking down more than 600 gene functions in L4 larvae did not report defects for *ippk-1*, *sqv-8*, or *vps-15*, but we have compared their mutant phenotypes to the reported categories (Green *et al.* 2011). While *sqv-8(or888*ts*)* mutants resemble the phenotype category that includes RNAi knockdown of *sqv-4*, which functions in the same glycosaminoglycan synthesis pathway as *sqv-8*, the mutant phenotypes we have found for *vps-15(or1235*ts*)* and *ippk-1(or1577*ts*)* do not match any of the reported categories in Green *et al.* (2011). The lack of any match to the reported phenotype categories may reflect difference degrees in loss of gene function due to RNAi *vs.* our mutant alleles, or indicate that additional gonad membrane defect categories remain to be discovered.

Our results suggest that *vps-15* and *ippk-1* are important during adult oogenesis and may contribute to the dynamic remodeling of membranes that must accompany the movement of oocyte precursors in the syncytial hermaphrodite gonad, and their eventual cellularization and enlargement prior to maturation and fertilization. Although the development of the adult membrane architecture of the syncytial hermaphrodite gonad has been the subject of some investigation (Green *et al.* 2011; Amini *et al.* 2014), very little is known about the processes that control membrane dynamics in adult worms during oogenesis. Our identification of TS alleles of essential genes required for maintenance of adult gonad membrane architecture may make the investigation of membrane dynamics during adult oogenesis more tractable.

To understand how *vps-15* and *ippk-1* influence membrane dynamics in the adult gonad will require further investigation. Examining the expression of these two proteins over time in live animals using fluorescent protein fusions to the endogenous loci may provide insights. It also will be interesting to examine membrane dynamics over time in immobilized live adults using fluorescent protein fusions that mark different membrane compartments and spinning disk or light sheet confocal microscopy in wild-type and in TS-EL mutants after temperature up-shifts.

Finally, although some of the genes we have identified (*rpl-7*, *crn-3*, *abtm-1*, *drp-1*, and *atx-2*) likely affect oogenesis only indirectly, conditional alleles of these essential loci should prove useful for investigating their different requirements, and the 10 causal TS mutations we identified are the first conditional alleles reported for all nine genes. Furthermore, the high-throughput identification of TS-EL mutations made possible by next-generation DNA sequencing methods now make it possible to more efficiently isolate conditional alleles of many more of the ∼2500 *C. elegans* genes known to be essential. A systematic effort to isolate large numbers of TS-EL mutations in *C. elegans* should provide a valuable resource for the detailed investigation of many different biological processes in this model organism.

## 

## Supplementary Material

Supporting Information
