# Supplementary material for: High-Throughput Cloning of Temperature-Sensitive Caenorhabditis elegans Mutants with Adult Syncytial Germline Membrane Architecture Defects
Source: G3 (Bethesda). 2015 Aug 26;5(11):2241–55. doi: 10.1534/g3.115.021451 (PMC4632044; doi:10.1534/g3.115.021451)
Supplement: Supporting Information [file supp_5_11_2241__index.html]

High-Throughput Cloning of Temperature-Sensitive Caenorhabditis elegans Mutants with Adult Syncytial Germline Membrane Architecture Defects — Supporting Information 

# High-Throughput Cloning of Temperature-Sensitive *Caenorhabditis elegans* Mutants with Adult Syncytial Germline Membrane Architecture Defects

## Supporting Information for Lowry *et al.*, 2015

**Files in this Data Supplement:**

- Supporting Information - Figures S1-S4 and Tables S1-S2 (PDF, 7 MB)
- Figure S1 - SNP Mapping Data for temperature-sensitive mutations without identified causal mutations. (PDF, 566 KB)
- Figure S2 - Adult germline defects in wild type and in *atx-2(or821*ts*)*, *crn-3(or959*ts*)*, *abtm-1(or1400*ts*)*, and *rpl-7(or990*ts*)* mutants following temperature up-shifts to the restrictive temperature (26°C) at the L1 and L4 larval stages. (PDF, 1 MB)
- Figure S3 - Adult germline defects in *drp-1(or1393*ts*)* and *sqv-8(or888*ts*)* mutants. (PDF, 4 MB)
- Figure S4 - Lack of germline defects in *drp-1(or1393*ts*)* and *sqv-8(or888*ts*)* mutants after young adults were up-shifted to the restrictive temperature (26°C). (PDF, 1 MB)
- Table S1 - Complementation test results that identified causal mutations in ten temperature-sensitive Osm/Ste mutants. (PDF, 35 KB)
- Table S2 - Strains used for complementation tests that identified causal mutations. (PDF, 28 KB)
