# Supplementary material for: High-Throughput Cloning of Temperature-Sensitive Caenorhabditis elegans Mutants with Adult Syncytial Germline Membrane Architecture Defects
Source: G3 (Bethesda). 2015 Aug 26;5(11):2241–55. doi: 10.1534/g3.115.021451 (PMC4632044; doi:10.1534/g3.115.021451)
Supplement: Supporting Information [file supp_g3.115.021451_021451SI.pdf]

G3 · Genes | Genomes | Genetics

High-Throughput Cloning of Temperature-Sensitive *Caenorhabditis elegans* Mutants with Adult Syncytial Germline Membrane Architecture Defects

Josh Lowry, John Yochem, Chien-Hui Chuang**,** Kenji Sugioka, Amy A. Connolly, and Bruce Bowerman

Institute of Molecular Biology, University of Oregon, Eugene, OR 97403

Corresponding Author: bbowerman@molbio.uoregon.edu

DOI: 10.1534/g3.115.021451

Figure S1

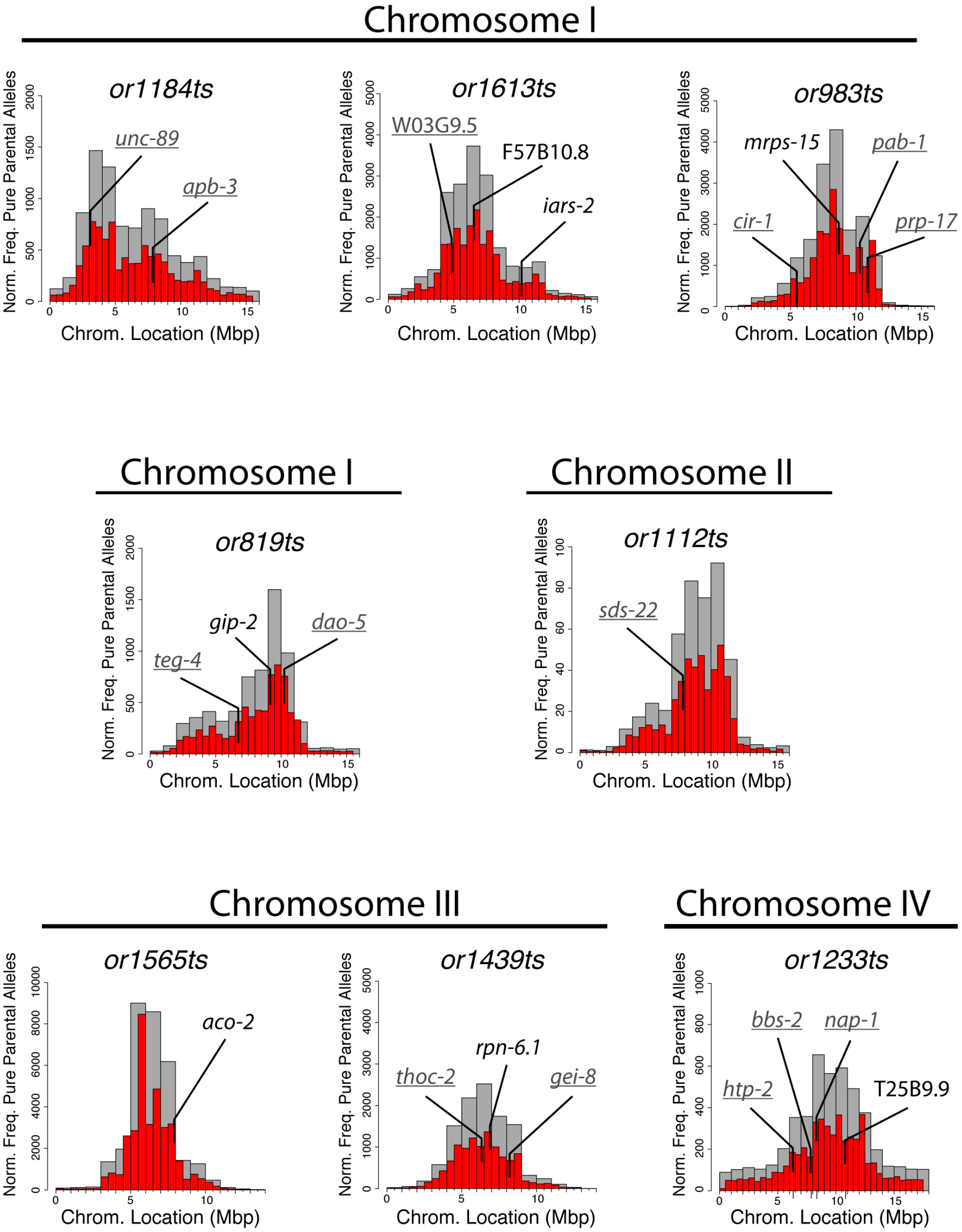

Figure S1 SNP Mapping Data for temperature-sensitive mutations without identified causal mutations. For each mutant, the frequency of homozygous parental alleles was plotted against chromosomal position, in bins of either one megabase (grey bars) or half-megabase (red bars). Gene names on each plot are essential genes in which missense mutations were detected. Underlined gene names are those for which complementation tests were performed; in all cases the mutations complemented each other (unpublished data).

Figure S2

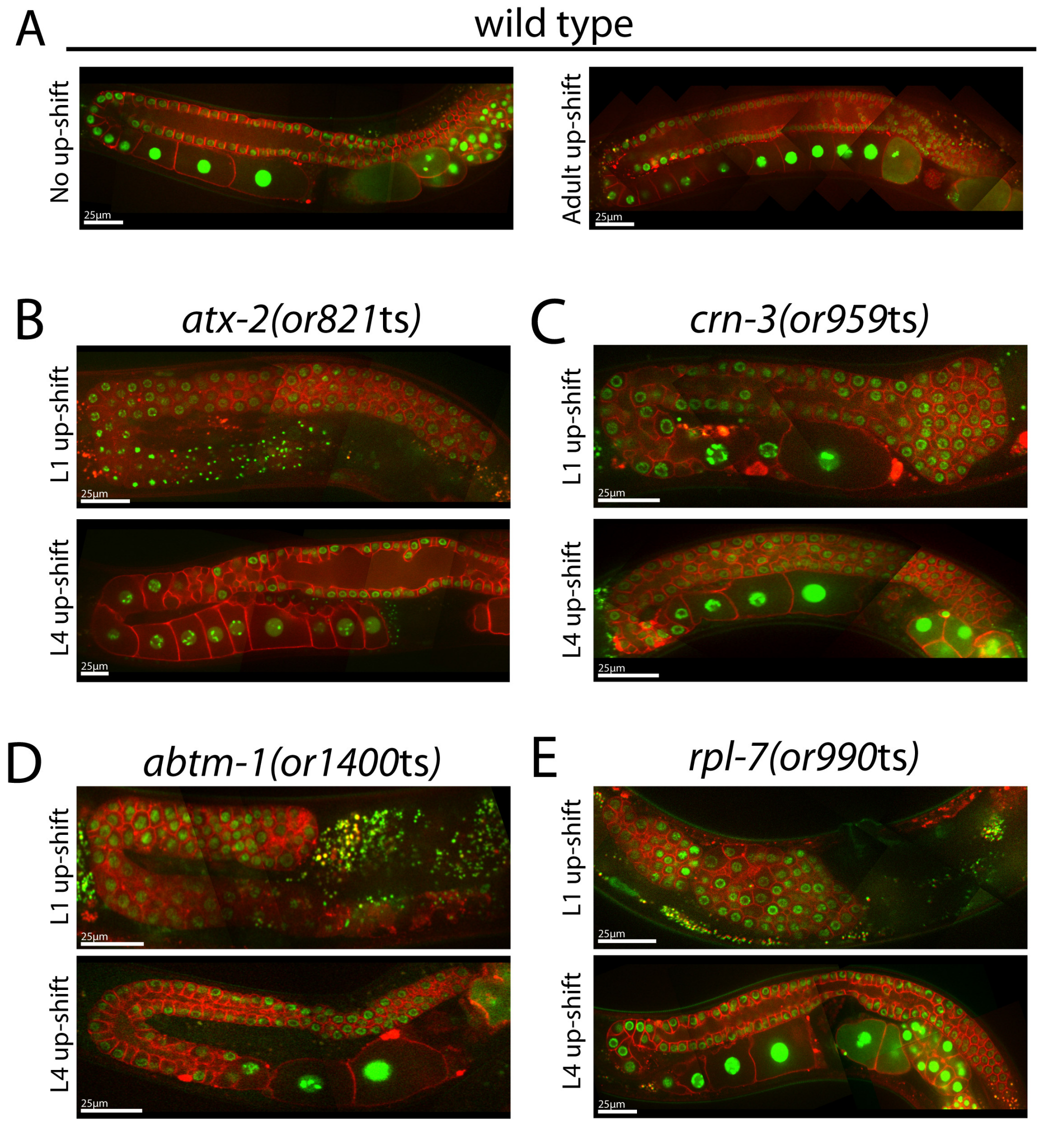

Figure S2 Adult germline defects in wild type and in *atx-2(or821*ts*)*, *crn-3(or959*ts*)*, *abtm-1(or1400*ts*)*, and *rpl-7(or990*ts*)* mutants following temperature up-shifts to the restrictive temperature (26°C) at the L1 and L4 larval stages. Composite images were prepared as previously described (Figure 3) for the adult hermaphrodite gonad in wild type (A) and mutants (B-E).

Figure S3

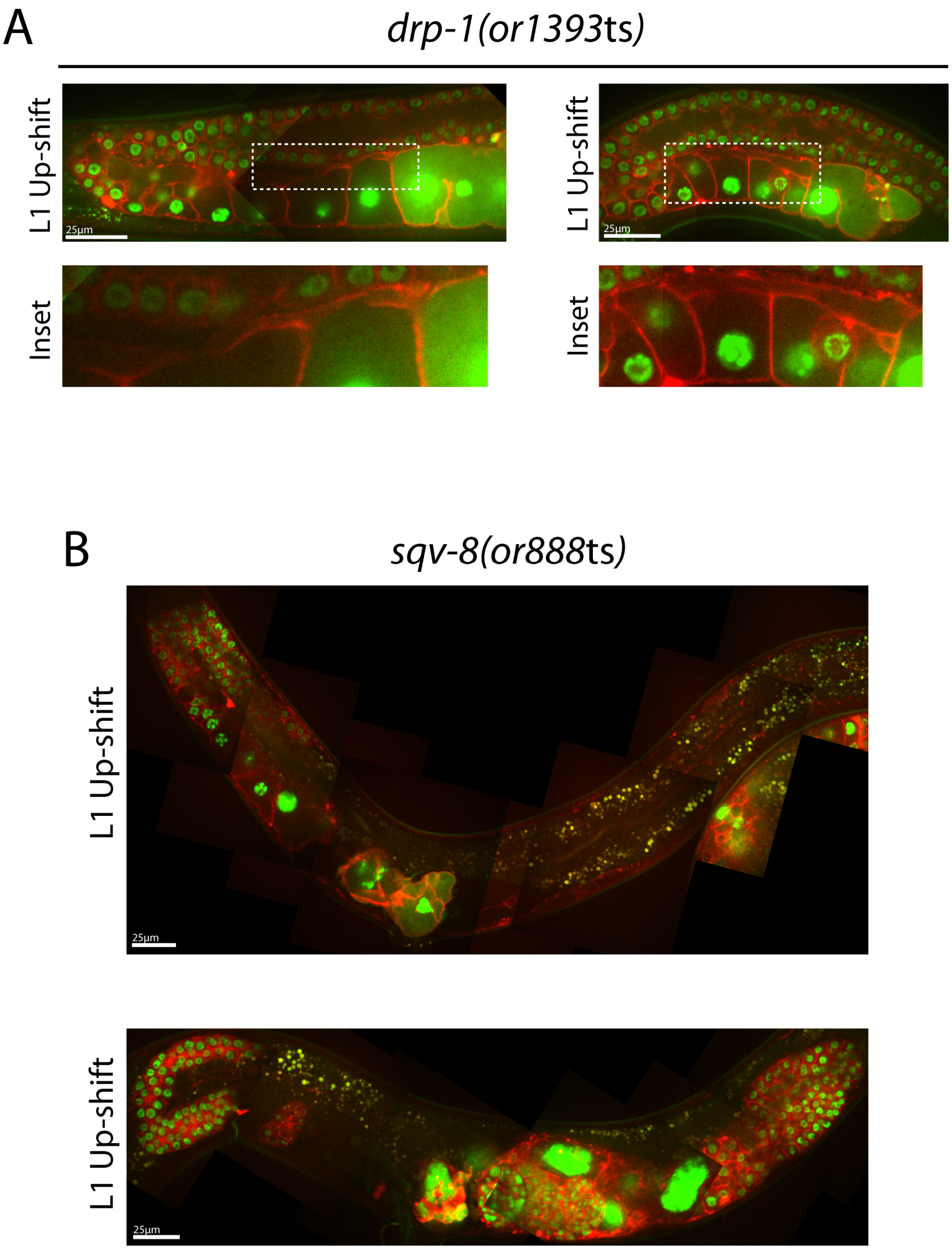

Figure S3 Adult germline defects in *drp-1(or1393*ts*)* and *sqv-8(or888*ts*)* mutants. (A) The rachis appears enlarged in the distal gonad and extends to the most mature oocytes in both examples. White boxes show the magnified region in the insets below. (B) Asymmetry of germline defects in *sqv-8(or888*ts*)*. In the upper panel, one arm is completely absent. In the lower panel left arm is small and appears not to extend to the uterus.

Figure S4

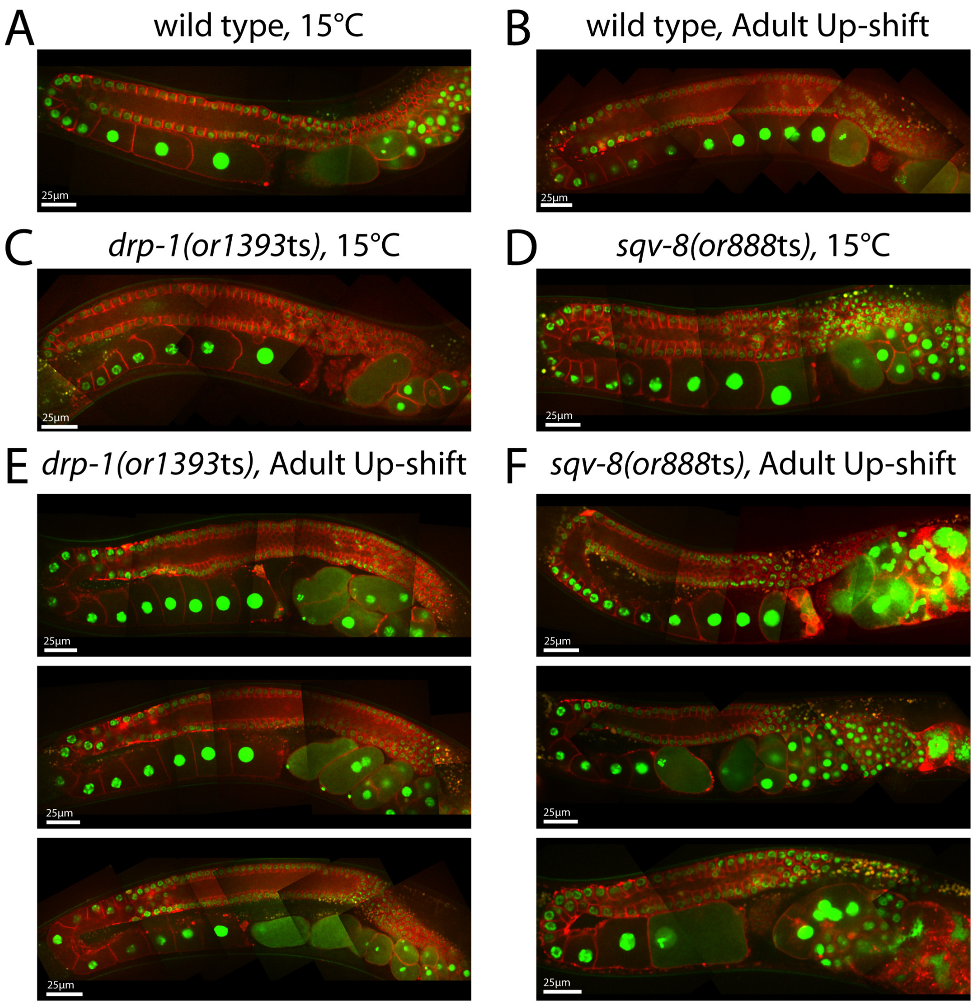

Figure S4 Lack of germline defects in *drp-1(or1393*ts*)* and *sqv-8(or888*ts*)* mutants after young adults were up-shifted to the restrictive temperature (26˚C). Composite images of the germline were prepared as previously described for wild type (A, B), *drp-1(or1393*ts*)* (C, E), and *sqv-8(or888*ts*)* (D, F) worms. These animals were either grown entirely at 15°C, or shifted to 26°C as young adults for 18 hours.

Table S1 Complementation test results that identified causal mutations in ten temperature-sensitive Osm/Ste mutants. Percent embryonic lethality was scored at 26°C for each genotype, followed by the number of embryos scored in parentheses.

Genotype	Embryonic Lethality 26°C	Genotype	Embryonic Lethality 26°C
*or821*ts	99.6% (715)	*or1235*ts	100% (117)
*or821*ts/+	8.3% (144)	*or1235*ts/+	3.6% (446)
*or821*ts/*atx-2(tm4373)*	98.8% (254)	*or1235*ts/*vps-15(ok3132)*	99.0% (517)
atx-2(tm4373)/+	13.2% (371)	*vps-15(ok3132)*/+	19.6% (255)
*or888*ts	99.7% (311)	*or1393*ts	99.7% (294)
*or888*ts/+	2.0% (250)	*or1393*ts/+	1.1% (363)
*or888*ts/*sqv-8(n2822)*	99.5% (198)	*or1393*ts/*drp-1(tm1108)*	88.2% (493)
sqv-8(n2822)/+	1.07% (280)	*drp-1(tm1108)*/+	4.8% (399)
		drp-1(tm1108)	90.1% (272)
*or959*ts	74.6% (405)		
*or959*ts/+	2.6% (288)	*or1400*ts	100% (181)
*or959*ts/*crn-3(ok2269)*	80.6% (139)	*or1400*ts/+	3.9% (442)
crn-3(ok2269)/+	8.2% (195)	*or1400*ts/*abtm-1(tm2721)*	100% (540)
crn-3(ok2269)	91.0% (167)		
		*or1572*ts	100% (465)
*or990*ts	93.8% (130)	*or1572*ts/+	1.1% (366)
*or990*ts/+	2.9% (450)	*or1572*ts/*ippk-1(tm4718)*	100% (247)
*or1247*ts	97.5% (81)	*ippk-1*(*tm4718*)/+	3.2% (342)
*or1247*ts/+	1.8% (325)		
*or990*ts/*or1247*ts	78.0% (91)		
*or1088*ts	84.9% (179)		
*or1088*ts/+	4.5% (220)		
*or1088*ts/*ndg-4(sa529)*	91.2% (239)		
ndg-4(sa529)/+	1.9% (266)		
ndg-4(sa529)	100%		

Table S2 Strains used for complementation tests that identified causal mutations. The mutations in these strains failed to complement the Osm/Ste mutations we tested (see Figure 1 and Table S1). No alleles are available for *rpl-7*, but our two TS Osm/Ste alleles failed to complement each other (see text). *Phenotype description from Shohei Motani (National Bio-Resource Project of the MEXT, Japan); +Phenotype description from the C. elegans Knockout Consortium.

Strain Name	Genotype	Allele Phenotype
FX02721	*abtm-1(tm2721)*/+ I	Let/Ste*
FX14556	+/hT2 I; *atx-2(tm4373)*/hT2 III	Let/Ste*
VC1715	*crn-3(ok2269)* II	Emb Let (Table S1)
EU2900	*drp-1(tm1108)* IV	Emb Let (Table S1; Lu et al, 2011)
FX14701	*ippk-1(tm4718)*/mIn1 II	Let/Ste*
JT529	*ndg-4(sa529)* III	Emb Let (Table S1)
MT7483	*sqv-8(n2822)*/mnC1 II	Emb Let (Herman et al, 1999)
VC2382	*vps-15(ok3132)*/mIn1 II	Larval arrest+
