# Supplementary material for: High-Throughput Cloning of Temperature-Sensitive Caenorhabditis elegans Mutants with Adult Syncytial Germline Membrane Architecture Defects
Source: G3 (Bethesda). 2015 Aug 26;5(11):2241–55. doi: 10.1534/g3.115.021451 (PMC4632044; doi:10.1534/g3.115.021451)
Supplement: Supporting Information [file supp_g3.115.021451_FigureS1.pdf]

Figure S1

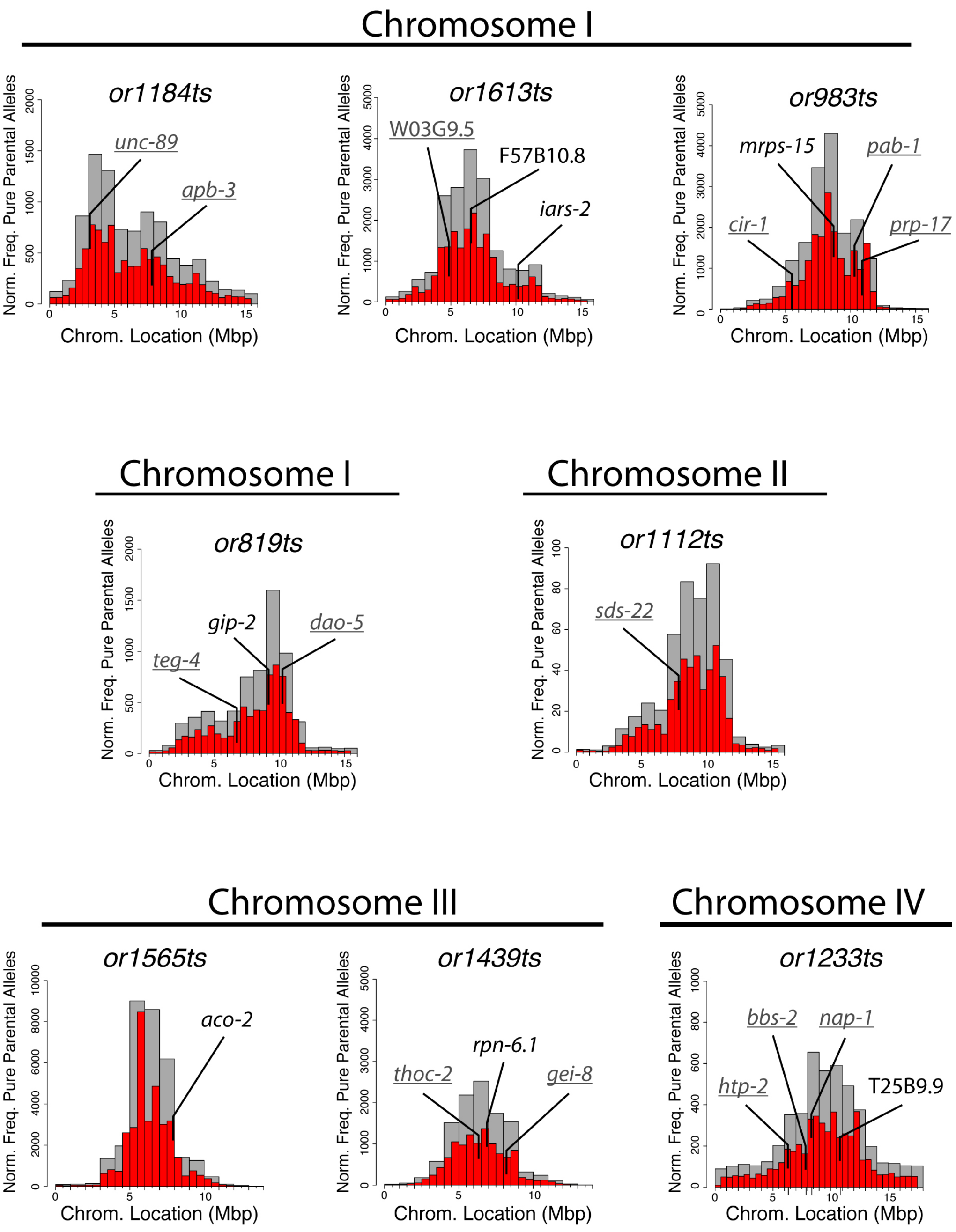

Figure S1 SNP Mapping Data for temperature-sensitive mutations without identified causal mutations. For each mutant, the frequency of homozygous parental alleles was plotted against chromosomal position, in bins of either one megabase (grey bars) or half-megabase (red bars). Gene names on each plot are essential genes in which missense mutations were detected. Underlined gene names are those for which complementation tests were performed; in all cases the mutations complemented each other (unpublished data).
