# Supplementary material for: High-Throughput Cloning of Temperature-Sensitive Caenorhabditis elegans Mutants with Adult Syncytial Germline Membrane Architecture Defects
Source: G3 (Bethesda). 2015 Aug 26;5(11):2241–55. doi: 10.1534/g3.115.021451 (PMC4632044; doi:10.1534/g3.115.021451)
Supplement: Supporting Information [file supp_g3.115.021451_FigureS2.pdf]

Figure S2

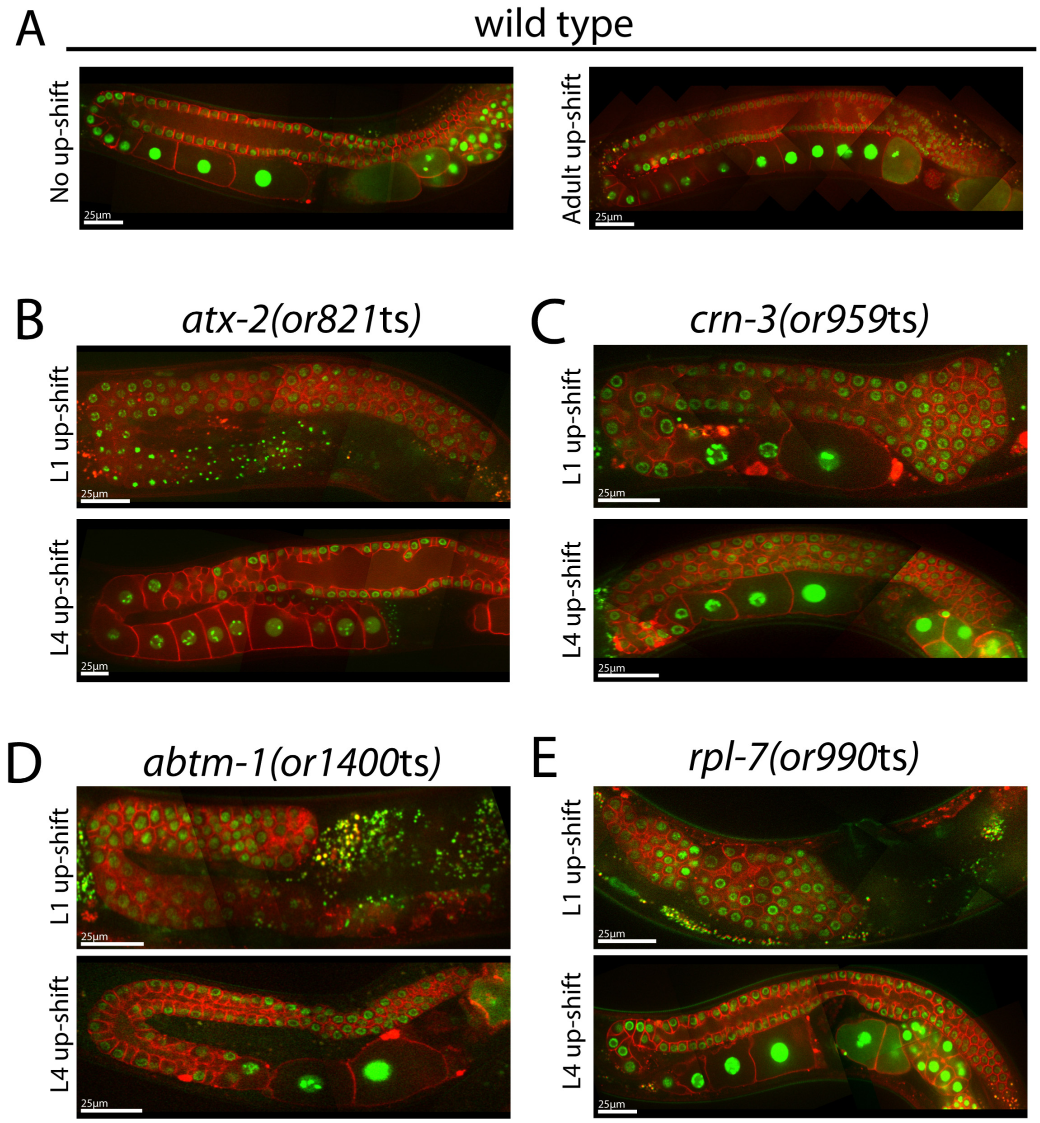

Figure S2 Adult germline defects in wild type and in *atx-2(or821*ts*)*, *crn-3(or959*ts*)*, *abtm-1(or1400*ts*)*, and *rpl-7(or990*ts*)* mutants following temperature up-shifts to the restrictive temperature (26°C) at the L1 and L4 larval stages. Composite images were prepared as previously described (Figure 3) for the adult hermaphrodite gonad in wild type (A) and mutants (B-E).
