# Supplementary material for: High-Throughput Cloning of Temperature-Sensitive Caenorhabditis elegans Mutants with Adult Syncytial Germline Membrane Architecture Defects
Source: G3 (Bethesda). 2015 Aug 26;5(11):2241–55. doi: 10.1534/g3.115.021451 (PMC4632044; doi:10.1534/g3.115.021451)
Supplement: Supporting Information [file supp_g3.115.021451_FigureS3.pdf]

Figure S3

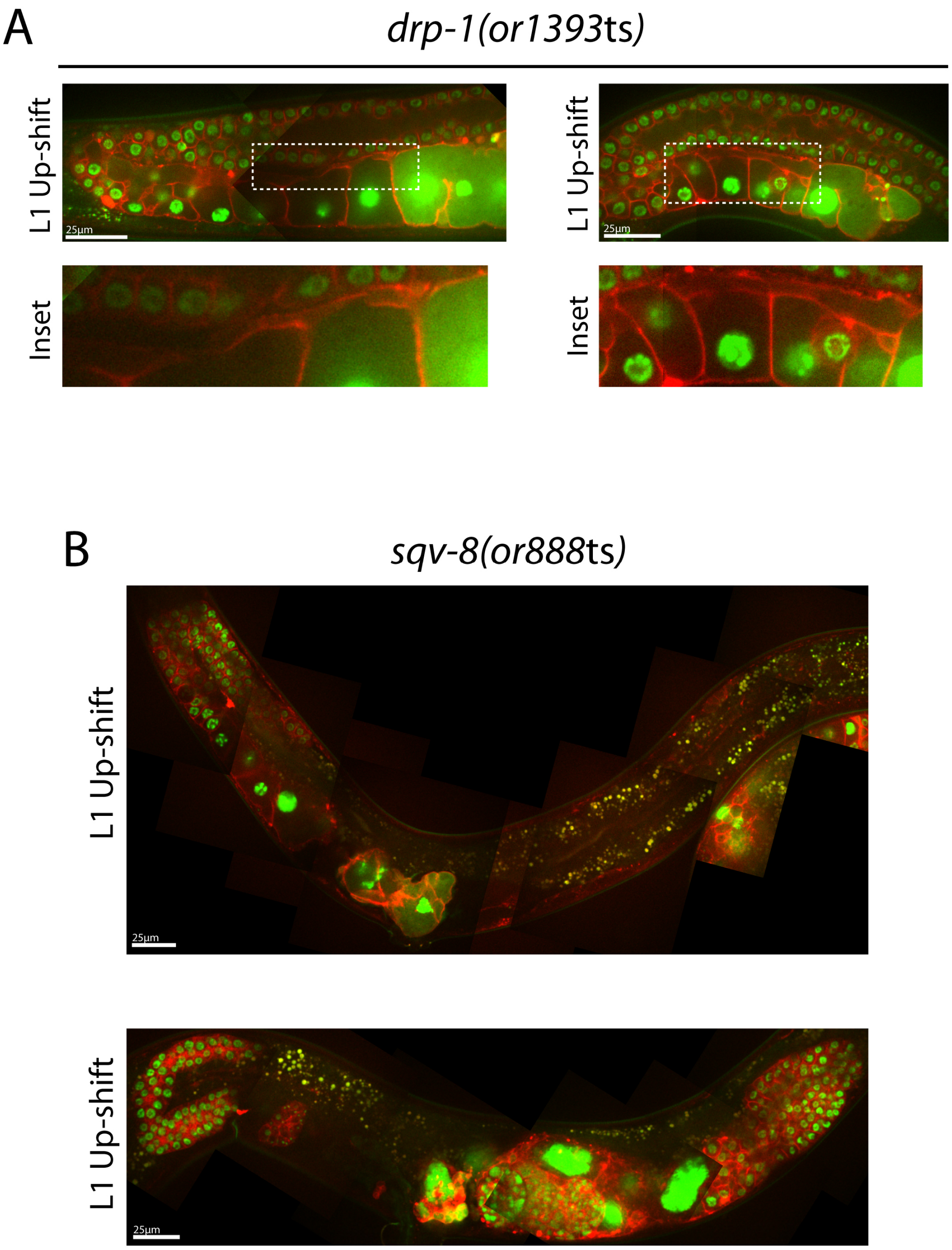

Figure S3 Adult germline defects in *drp-1(or1393*ts*)* and *sqv-8(or888*ts*)* mutants. (A) The rachis appears enlarged in the distal gonad and extends to the most mature oocytes in both examples. White boxes show the magnified region in the insets below. (B) Asymmetry of germline defects in *sqv-8(or888*ts*)*. In the upper panel, one arm is completely absent. In the lower panel left arm is small and appears not to extend to the uterus.
