# Supplementary material for: High-Throughput Cloning of Temperature-Sensitive Caenorhabditis elegans Mutants with Adult Syncytial Germline Membrane Architecture Defects
Source: G3 (Bethesda). 2015 Aug 26;5(11):2241–55. doi: 10.1534/g3.115.021451 (PMC4632044; doi:10.1534/g3.115.021451)
Supplement: Supporting Information [file supp_g3.115.021451_FigureS4.pdf]

Figure S4

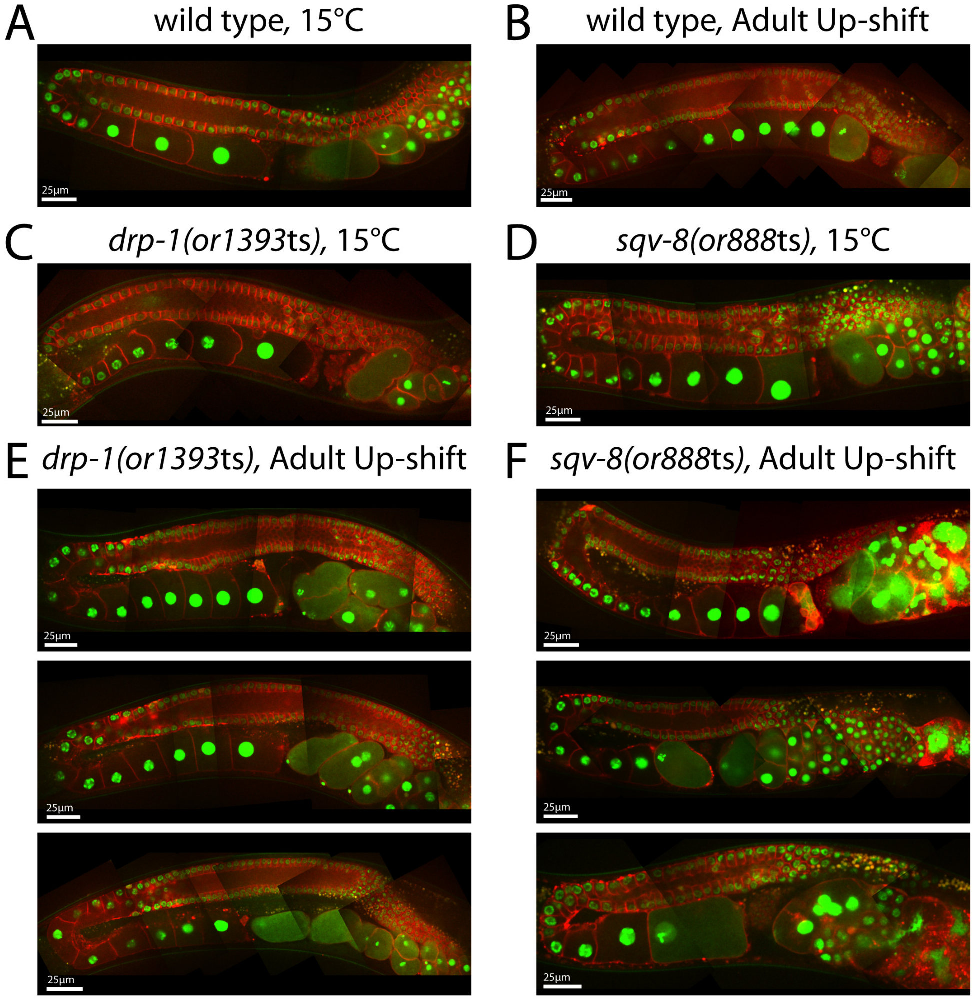


**Figure S4** Lack of germline defects in *drp-1(or1393*ts*)* and *sqv-8(or888*ts*)* mutants after young adults were up-shifted to the restrictive temperature (26˚C). Composite images of the germline were prepared as previously described for wild type (A, B), *drp-1(or1393*ts*)* (C, E), and *sqv-8(or888*ts*)* (D, F) worms. These animals were either grown entirely at 15°C, or shifted to 26°C as young adults for 18 hours.
