# Supplementary material for: High-Throughput Cloning of Temperature-Sensitive Caenorhabditis elegans Mutants with Adult Syncytial Germline Membrane Architecture Defects
Source: G3 (Bethesda). 2015 Aug 26;5(11):2241–55. doi: 10.1534/g3.115.021451 (PMC4632044; doi:10.1534/g3.115.021451)
Supplement: Supporting Information [file supp_g3.115.021451_TableS1.pdf]

Table S1 Complementation test results that identified causal mutations in ten temperature-sensitive Osm/Ste mutants. Percent embryonic lethality was scored at 26°C for each genotype, followed by the number of embryos scored in parentheses.

Genotype	Embryonic Lethality 26°C
*or821*ts	99.6% (715)
*or821*ts/+	8.3% (144)
*or821*ts/*atx-2(tm4373)*	98.8% (254)
atx-2(tm4373)/+	13.2% (371)
*or888*ts	99.7% (311)
*or888*ts/*sqv-8(n2822)*	99.5% (198)
*or888*ts/+	2.0% (250)
sqv-8(n2822)/+	1.07% (280)
*or959*ts	74.6% (405)
*or959*ts/+	2.6% (288)
*or959*ts/*crn-3(ok2269)*	80.6% (139)
crn-3(ok2269)/+	8.2% (195)
crn-3(ok2269)	91.0% (167)
*or990*ts	93.8% (130)
*or990*ts/+	2.9% (450)
*or1247*ts	97.5% (81)
*or1247*ts/+	1.8% (325)
*or990*ts/*or1247*ts	78.0% (91)
*or1088*ts	84.9% (179)
*or1088*ts/+	4.5% (220)
*or1088*ts/*ndg-4(sa529)*	91.2% (239)
ndg-4(sa529)/+	1.9% (266)
ndg-4(sa529)	100%

Genotype	Embryonic Lethality 26°C
*or1235*ts	100% (117)
*or1235*ts/+	3.6% (446)
*or1235*ts/*vps-15(ok3132)*	99.0% (517)
vps-15(ok3132)/+	19.6% (255)
*or1393*ts	99.7% (294)
*or1393*ts/+	1.1% (363)
*or1393*ts/*drp-1(tm1108)*	88.2% (493)
drp-1(tm1108)/+	4.8% (399)
drp-1(tm1108)	90.1% (272)
*or1400*ts	100% (181)
*or1400*ts/+	3.9% (442)
*or1400*ts/*abtm-1(tm2721)*	100% (540)
*or1572*ts	100% (465)
*or1572*ts/+	1.1% (366)
*or1572*ts/*ippk-1(tm4718)*	100% (247)
ippk-1(*tm4718*)/+	3.2% (342)
