# Supplementary material for: High-Throughput Cloning of Temperature-Sensitive Caenorhabditis elegans Mutants with Adult Syncytial Germline Membrane Architecture Defects
Source: G3 (Bethesda). 2015 Aug 26;5(11):2241–55. doi: 10.1534/g3.115.021451 (PMC4632044; doi:10.1534/g3.115.021451)
Supplement: Supporting Information [file supp_g3.115.021451_TableS2.pdf]

Table S2 Strains used for complementation tests that identified causal mutations. The mutations in these strains failed to complement the Osm/Ste mutations we tested (see Figure 1 and Table S1). No alleles are available for *rpl-7*, but our two TS Osm/Ste alleles failed to complement each other (see text). *Phenotype description from Shohei Motani (National Bio-Resource Project of the MEXT, Japan); +Phenotype description from the C. elegans Knockout Consortium.

Strain Name	Genotype	Allele Phenotype
FX02721	*abtm-1(tm2721)*/+ I	Let/Ste*
FX14556	+/hT2 I; *atx-2(tm4373)*/hT2 III	Let/Ste*
VC1715	*crn-3(ok2269)* II	Emb Let (Table S1)
EU2900	*drp-1(tm1108)* IV	Emb Let (Table S1; Lu et al, 2011)
FX14701	*ippk-1(tm4718)*/mIn1 II	Let/Ste*
JT529	*ndg-4(sa529)* III	Emb Let (Table S1)
MT7483	*sqv-8(n2822)*/mnC1 II	Emb Let (Herman et al, 1999)
VC2382	*vps-15(ok3132)*/mIn1 II	Larval arrest+
